# Pan-RAF Inhibition Shows Anti-Leukemic Activity in *RAS*-Mutant Acute Myeloid Leukemia Cells and Potentiates the Effect of Sorafenib in Cells with *FLT3* Mutation

**DOI:** 10.3390/cancers12123511

**Published:** 2020-11-25

**Authors:** Joseph D. Khoury, Mehrnoosh Tashakori, Hong Yang, Sanam Loghavi, Ying Wang, Jing Wang, Sujan Piya, Gautam Borthakur

**Affiliations:** 1 Department of Hematopathology, The University of Texas MD Anderson Cancer Center, 1515 Holcombe Boulevard, MS-072, Houston, TX 77030, USA; mtashakori@mdanderson.org (M.T.); hyang@mdanderson.org (H.Y.); SLoghavi@mdanderson.org (S.L.); 2 Department of Bioinformatics and Computational Biology, The University of Texas MD Anderson Cancer Center, Houston, TX 77030, USA; YWang31@mdanderson.org (Y.W.); jingwang@mdanderson.org (J.W.); 3 Department of Leukemia, The University of Texas MD Anderson Cancer Center, 1515 Holcombe Boulevard, MS-072, Houston, TX 77030, USA; SPiya@mdanderson.org

**Keywords:** acute myeloid leukemia, RAF, RAS, FLT3, preclinical

## Abstract

**Simple Summary:**

We demonstrate that the pan-RAF inhibitor LY3009120 induces apoptosis and inhibits proliferation in AML cells harboring *RAS* or *FLT3* mutations through action on the RAS/RAF/MEK/ERK and the AKT/mTOR pathways. Notably, pan-RAF inhibition combined with Ara-C overcomes drug resistance mediated by bone marrow-derived mesenchymal stem cells. Furthermore, the combination of LY3009120 and tyrosine kinase inhibition with sorafenib appears to synergistically increase apoptosis in AML cells carrying *FLT3*-ITD mutation.

**Abstract:**

RAF molecules play a critical role in cell signaling through their integral impact on the RAS/RAF/MEK/ERK signaling pathway, which is constitutively activated in a sizeable subset of acute myeloid leukemia (AML) patients. We evaluated the impact of pan-RAF inhibition using LY3009120 in AML cells harboring mutations upstream and downstream of RAF. LY3009120 had anti-proliferative and pro-apoptotic effects and suppressed pERK1/2 levels in leukemic cells with *RAS* and *FLT3* mutations. Using reverse protein phase array analysis, we identified reductions in the expression/activation of cell signaling components downstream of RAF (activated p38) and cell cycle regulators (Wee1/cyclin B1, Cdc2/Cdk1, activated Rb, etc.). Notably, LY3009120 potentiated the effect of Ara-C on AML cells and overcame bone marrow mesenchymal stromal cell-mediated chemoresistance, with *RAS*-mutated cells showing a notable reduction in pAKT (Ser473). Furthermore, the combination of LY3009120 and sorafenib resulted in significantly higher levels of apoptosis in AML cells with heterozygous and hemizygous *FLT3* mutations. In conclusion, pan-RAF inhibition in AML using LY3009120 results in anti-leukemic activity, and combination with Ara-C or sorafenib potentiates its effect.

## 1. Introduction

The RAS/RAF/MEK/ERK (MAPK) signaling pathway plays a critical role in the transmission of proliferative signals from extracellular stimuli to downstream effectors. Pathway activation is mediated physiologically by ligand binding and receptor tyrosine kinase activation resulting in a cascade of kinases being activated leading to downstream phosphorylation of extracellular signal-regulated kinase 1/2 (ERK1/2) on residues Thr202 and Tyr204 [1,2]. Active, phosphorylated ERK (pERK1/2) results in transactivation of transcription factors and gene expression modulations that collectively promote cell survival, differentiation, and proliferation [1,2]. Constitutive activation of the MAPK pathway results from gain-of-function mutations in genes encoding pathway constituents, particularly *NRAS*, a common finding in up to 11% of patients with acute myeloid leukemia (AML) [3,4,5]. In another sizeable subset of AML patients, constitutive ERK activation is due to gain-of-function tyrosine kinase domain mutations in upstream signaling molecules, particularly *FLT3* [6].

Direct targeting of oncogenic RAS proteins in myeloid malignancies has not been feasible clinically thus far; however, multiple agents targeting upstream and/or downstream components of the pathway have been developed. Most notable among the former are kinases inhibitors that target FLT3, which show a consistent reduction in pERK levels in the absence of emergent resistance [7,8]. ERK activation was observed in *FLT3*-mutated AML cells at relapse, suggesting that MAPK pathway activation persists even when FLT3 phosphorylation is suppressed [9]. Efforts to target downstream kinases have focused on RAF, MEK, and ERK, almost exclusively in neoplasms with wild-type *RAS*. Clinical trials evaluating the efficacy of MEK1/2 inhibitors in AML have shown promising albeit mixed results [10,11,12]. To circumvent compensatory resistance through upregulation of AKT/mTOR signaling pathways, a combination of MEK and AKT inhibitors was evaluated by Ragon et al. but showed a lack of clinical efficacy [10]. Concomitant inhibition of FLT3 and ERK activation seems to result in a substantial effect on leukemic cells with *FLT3*-ITD in vitro [7]. Notably, recent data from McMahon et al. indicate that MAPK pathway activation, mainly through clonal acquisition of *NRAS* mutations, represents a major dynamic resistance mechanism to mutation-selective tyrosine kinase inhibitor therapy in AML [13]. Against a backdrop of limited efficacy from MEK inhibition [14] and a need to suppress pathway activation, particularly in view of current considerations for including FLT3 inhibitors to frontline AML treatment regimens [15], there is a continued need to explore novel and potent pathway inhibitors.

ERK signaling requires RAS-induced RAF (ARAF, BRAF, and CRAF) homodimerization and heterodimerization [16]. Specific RAF inhibitors such as the BRAF V600E/K inhibitors vemurafenib and dabrafenib induce paradoxical hyperactivation of wild-type RAF in normal and neoplastic cells with upregulation of downstream pERK1/2 signaling [17,18,19]. In a disease such as AML where activating mutations involving RAF genes are exceedingly rare, this has effectively excluded investigations into the utility of RAF inhibition to date. However, new RAF inhibitors targeting both monomeric and dimeric RAF molecules have provided a novel therapeutic approach. LY3009120, a third-generation RAF inhibitor, equipotentially inhibits monomeric as well as dimeric forms of each of the three members of the RAF protein family [20,21]. LY3009120 works by stably occupying both promoters of RAF dimerization, and—unlike vemurafenib—has been shown to have minimal paradoxical activation while being effective in the setting of mutant *RAS* or oncogenic *BRAF* deletions [20,21,22]. These properties present potential value in AML therapy.

In this study, we tested the effect of LY3009120 on AML cells harboring mutant *RAS* or *FLT3*. We then explored whether LY3009120 has the potential to overcome bone marrow stroma-induced chemoresistance and examined its activity in combination with sorafenib.

## 2. Materials and Methods

### 2.1. Reagents and Antibodies

LY3009120 was provided by Eli Lilly and Company (Indianapolis, IN, USA). Antibodies against pERK1/2 (Thr202/Tyr204), ERK1/2, phosphorylated Akt (Ser473) (pAKT), phosphorylated P70S6 kinase (Thr421/Ser424) (p-P70S6K), P70S6 kinase, phosphorylated S6 ribosomal protein (Ser235/236) (pS6), S6 ribosomal protein (5G10), elF4E, and β-actin were purchased from Cell Signaling Technology (Danvers, MA, USA); antibody against FLT3 (S18) was purchased from Santa Cruz Biotechnology (Dallas, TX, USA).

### 2.2. Cell Lines, Co-Culture Experiments, and Pharmacologic Reagents

The human AML cell lines OCI-AML3, MOLM13, and MV4-11 were kindly provided by Dr. Marina Konopleva (MD Anderson Cancer Center). The identity of all cell lines was validated by short tandem repeat DNA fingerprinting using the AmpFISTR identifier kit according to manufacturer’s instructions (Applied Biosystems). All cell lines were maintained at 37 °C with 5% CO_2_ in a humidified incubator in a culture medium consisting of RPMI-1640 (Sigma Aldrich, St. Louis, MO, USA) supplemented with 10% heat-inactivated fetal bovine serum (FBS) (Atlanta Biologicals, Flowery Branch, GA, USA), penicillin, and streptomycin.

Co-culture experiments were performed by incubating AML cells with a feeder layer of human mesenchymal stem cells (MSC) derived from normal bone marrow samples in accordance with institutional guidelines. MSC cultures were plated for 24 h at 37 °C with 5% CO_2_ in a culture medium consisting of 20% FBS (Fisher Scientific, Hanover Park, IL, USA), α-MEM (Corning, Manassas, VA, USA) and 4% Human Platelet Lysate (EMD Millipore Corp, Billerica, MA, USA). Thereafter, the culture medium was removed and AML cells (OCI/AML3) were seeded on top of the MSC layer in a ratio of 4:1 (OCI/AML3:MSC) in RPMI-1640 and 10% FBS. Co-cultured cells were kept for 24 h at 37 °C with 5% CO_2_ before treatment (see below).

### 2.3. Cell Proliferation Assay

Cell viability was measured using the MTS ([3-(4,5-dimethylthiazol-2-yl)-5-(3-carboxymethoxyphenyl)-2-(4-sulfophenyl)-2h-tetrazolium]) assay (Abcam) per the manufacturer’s recommendations. Cell lines were cultured in triplicate in flat-bottomed 96-well plates at a concentration of 3–5 × 10^4^ cells/well. After cells were incubated with different concentrations ranging from 1 to 1000 nmol/L (nM) of LY3009120 for 24−120 h, 20 μL of MTS was added to each well for 4 h. Light absorbance (490 nm) levels of treated and untreated cells were then measured using a microplate reader to determine the optical density (OD490). MTS cell viability assays were conducted in triplicate on post-treatment samples. Cell growth inhibition was calculated using the following formula: growth inhibition (%) = [1 − (OD490 sample/OD490 control)] × 100.

### 2.4. Apoptosis and Cell Viability Assays

Apoptosis assessment was based on detection of annexin V expression using multiparameter flow cytometry (MFC) and an antibody conjugated to fluorescein isothiocyanate (FITC) (Invitrogen, Eugene, OR, USA). Briefly, 1 × 10^5^ cells were washed once with phosphate-buffered saline (PBS) and once with annexin V-binding buffer before incubation for 15 min with FITC-conjugated annexin V antibody in 100 µL annexin-binding buffer. Cells were then washed and analyzed on a Gallios flow cytometer (Beckman Coulter; Brea, CA, USA). Count Bright Beads (Molecular Probes, Grand Island, NY, USA) and counterstaining with DAPI were used to determine the number of positive cells and the absolute number of cells, respectively. Cell viability was determined based on staining with 4′,6-diamidino-2-phenylindole (DAPI) (Sigma-Aldrich, St. Louis, MO, USA).

### 2.5. Western Blot Assays

Cells were washed in PBS and lysed in cell lysis buffer (Cell Signaling) in the presence of 1 mM PMSF (Cell Signalling), and protein concentrations were determined using the bicinchoninic acid (BCA) protein assay (Pierce, Rockford, IL, USA). Equal amounts of protein were subjected to sodium dodecyl sulfate-polyacrylamide gel electrophoresis and transferred to polyvinylidene difluoride (PVDF) membranes (Bio-Rad, Hercules, CA, USA). β-actin was used as a loading control in all experiments. All assays were performed in replicate. 

### 2.6. Reverse Protein Phase Array (RPPA) Analysis

Cell lysates were serially diluted two-fold for five dilutions (from undiluted to 1:16 dilution) and arrayed on nitrocellulose-coated slides in an 11 × 11 format. Samples were probed with 305 antibodies by a tyramide-based signal amplification approach and visualized by 3,3’-diaminobenzidine colorimetric reaction. Slides were scanned on a flatbed scanner to produce 16-bit tiff images. Spots from tiff images were identified and the density was quantified by Array-Pro Analyzer. Relative protein levels for each sample were determined by interpolation of each dilution curve from the “standard curve” (supercurve) of the slide (antibody).

### 2.7. Statistical Analyses

All experiments were conducted in triplicate and results expressed as mean ± standard deviation (error bars). Statistical differences between groups were determined using paired Student’s *t*-test. The Chou–Talalay method was used to determine combination indices (CIs) for combination treatments (assuming mutual exclusivity); a CI value of 1 indicates an additive effect, <1 indicates synergism, and >1 indicates antagonism. The mean combination index (CI) values were calculated at different effect levels (50%, 75%, and 90% effective concentrations). Processing and statistical analysis of RPPA data were performed in R. Data were normalized using sample-by-sample median centering. The proteins in the heatmap were clustered using Ward’s clustering based on Pearson’s correlation. A *p*-value < 0.05 was considered statistically significant.

## 3. Results

### 3.1. LY3009120 Induces Anti-Leukemia Effects in AML Cells Harboring NRAS or FLT3 Mutations

**Figure 1:**
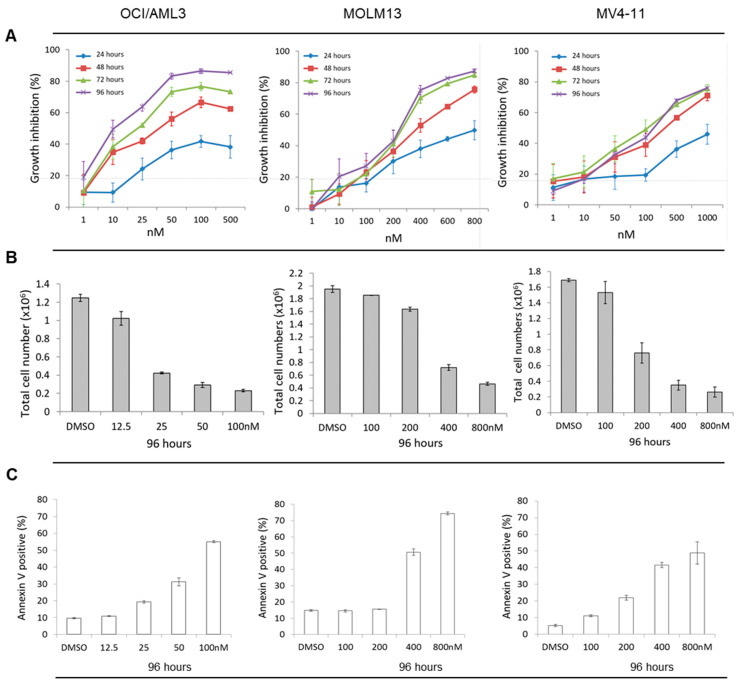
Optimization of LY3009120 treatment against acute myeloid leukemia (AML) cell lines. (**A**) Growth inhibition induced in OCI/AML3, MOLM13, and MV4-11 cell lines was measured following incubation with different doses of LY3009120 for 24, 48, 72 or 96 h. Treatment with LY3009120 for 96 h resulted in (**B**) significant cell death with a reduction in the number of AML cells and (**C**) induction of apoptosis as assessed by annexin V expression. [DMSO: dimethyl sulfoxide].

We investigated the antileukemic effect of LY3009120 on AML cell lines harboring activating mutations upstream and downstream of RAF resulting in constitutive activation of MAPK signaling. Cell lines used included OCI/AML3 with *NRAS*^Q61L^ mutation, MV4-11 with homozygous *FLT3*-ITD mutations, and MOLM13 with heterozygous *FLT3*-ITD mutation [23]. We first surveyed the antileukemic impact of LY3009120 on OCI/AML3, MOLM13, and MV4-11 at various concentrations and different incubation times. All cell lines showed time- and dose-dependent growth inhibition, cell death, and apoptosis, with the most notable effect being on OCI/AML3 cells treated for 96 h. (Figure 1A–C) These results demonstrate that LY3009120 has antiproliferative and pro-apoptotic effects on leukemic cells with *NRAS* and *FLT3*-ITD mutations, with a higher sensitivity in OCI/AML3, which harbors mutant *NRAS* and wild-type *FLT3*.

### 3.2. LY3009120 Impacts the RAS-Mediated Signaling Pathway in a Leukemia Cell-Specific Manner

**Figure 2:**
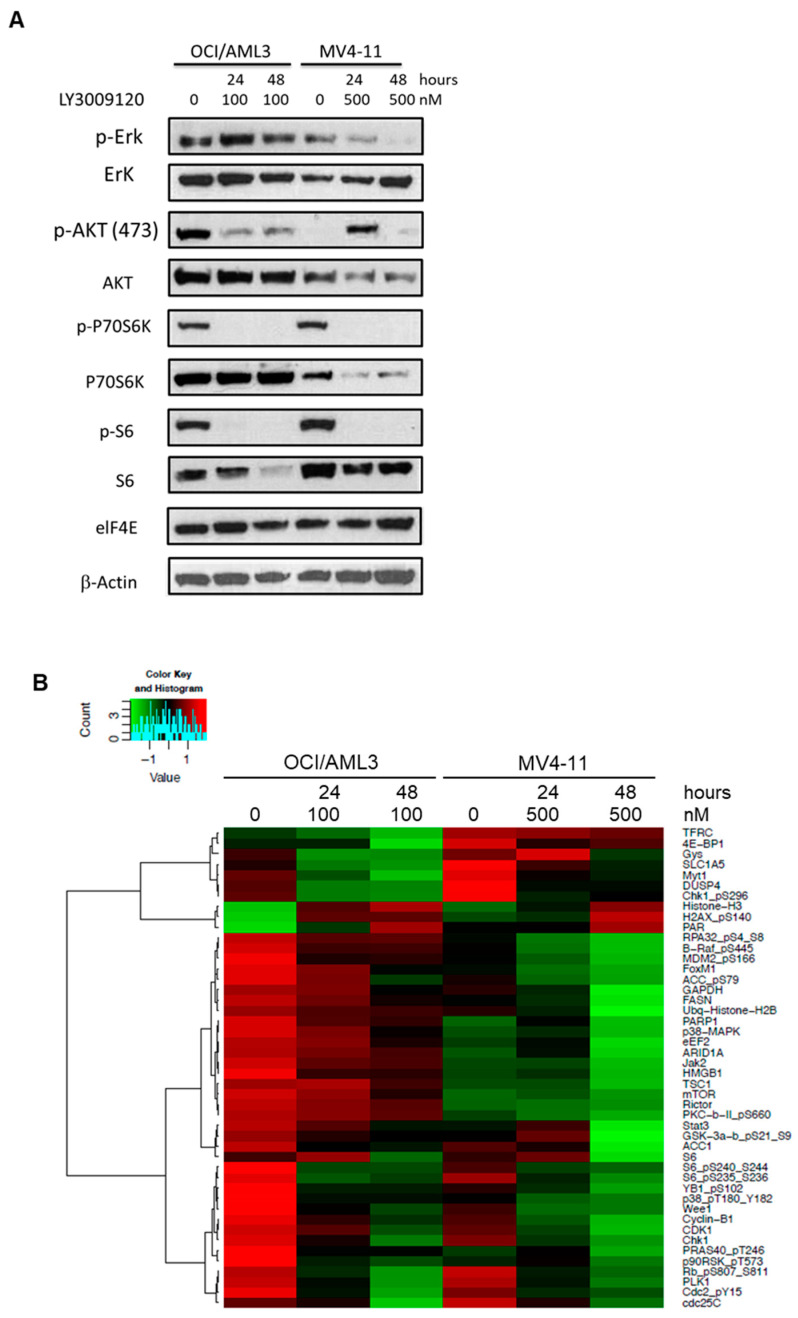
Impact of LY3009120 on cell signaling pathways in acute myeloid leukemia cells. (**A**) Immunoblots showing levels of phosphorylated and total ERK, AKT, P70S6K, and S6 proteins following pan-RAF inhibition. (**B**) Heatmap of reverse protein phase array evaluation depicting proteins with an absolute log_2_ expression level fold change > 0.8 (48-h exposure vs. baseline).

To determine the effect of LY3009120 on RAF-mediated signaling, we examined the expression and activation status of the pathway effector pERK1/2 as well as downstream targets and their possible cross-talk with other cell signaling pathways. Based on our growth inhibition and apoptosis data, we conducted this experiment using 100 nM and 500 nM concentrations of LY3009120 for OCI/AML3 and MV4-11 cells, respectively. LY3009120 treatment for 24 and 48 h suppressed pERK1/2 levels in MV4-11 in a time-dependent manner, with a much less pronounced effect in OCI/AML3. (Figure 2A) LY3009120 treatment resulted in a notable reduction in the pAKT level in OCI/AML3, while in MV4-11 upregulation in AKT activation was observed after 24-h but not after 48-h exposure. There was a clear reduction in pP70S6K and pS6 levels in both cell lines, while levels of elF4E remained unchanged. These findings suggest that LY3009120 has a potent effect on ERK activation in the setting of *FLT3*-ITD, whereas in the setting of a gain-of-function *RAS* mutations its impact might be dependent on cross-talks with inhibitory pathways as described previously [22] in other cell lines.

To assess the impact of LY3009120 on cell signaling pathways in AML cells, we treated OCI/AML3 and MV4-11 cells for 24 and 48 h, following which we analyzed cell lysates with RPPA to determine differences in protein expression and/or activation. Proteins with the highest levels of expression difference between baseline and 48-h exposure are summarized in Figure 2B. Given the biologic differences between OCI-AML3 and MV4-11 cells, they showed expected differing expression patterns particularly after 48 h of treatment with LY3009120. However, interestingly, there was a notable reduction in the expression/activation of components downstream of RAF (e.g., activated p38) and cell cycle regulators (e.g., Wee1/cyclin B1, Cdc2/Cdk1, activated Rb) in both cell lines. The full RPPA dataset is provided in Table S1.

### 3.3. Combining LY3009120 with Ara-C Overcomes Bone Marrow Stroma-Mediated Chemoresistance

**Figure 3:**
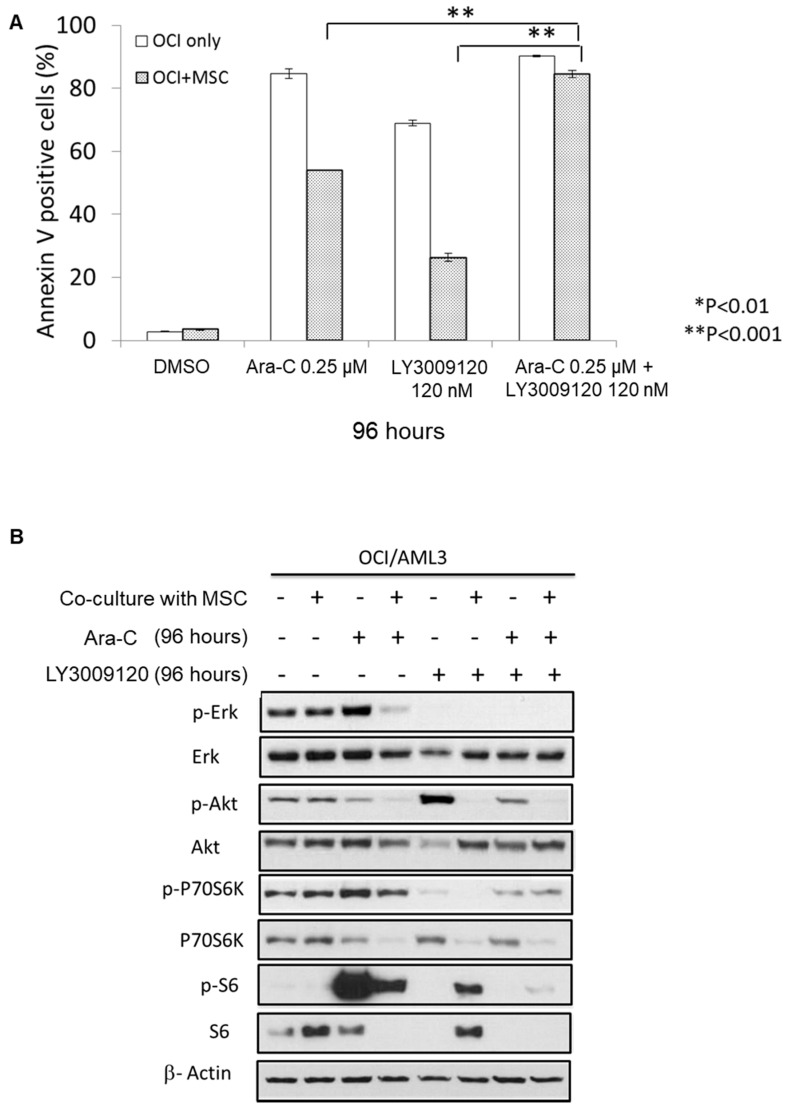
(**A**) Co-culture of OCI/AML3 cells with human bone marrow mesenchymal stem cells (MSC) had a protective effect against Ara-C and LY3009120 as single agents. In combination, the drugs had a synergistic effect with abrogation of MSC protection. [** *p* < 0.001] (**B**) Levels of phosphorylated and total ERK, AKT, P70S6K, and S6 proteins were determined by immunoblotting after treatment of OCI/AML3 with Ara-C (0.25 µM), LY3009120 (120 nM), or a combination of the two drugs, with or without MSC.

The bone marrow microenvironment has been shown to provide a protective effect for leukemic cells against various therapeutic agents [24,25]. To mimic the bone marrow microenvironment in vitro, we co-cultured OCI-AML3 cells on a supportive layer of MSC derived from the bone marrow of healthy donors [25]. OCI-AML3 cells treated with Ara-C alone in the presence of MSCs had significantly lower levels of apoptosis than control OCI-AML3 cells exposed to the same level of the drug. Exposure to LY3009120 alone demonstrated a similar pattern, with MSCs providing a protective antiapoptotic effect. Notably, combining Ara-C and LY3009120 resulted in significant mitigation of the protective effect of co-cultured MSCs. Namely, the combination of Ara-C (0.25 µM) and LY3009120 (120 nM) for 96 h induced a significantly higher percentage of apoptosis (85%) than Ara-C (54%) or LY3009120 (26%) alone in OCI/AML3 cells co-cultured with MSCs. (Figure 3A) A similar effect was detected with the same combination using lower doses of LY3009120 down to 20 nM. These data show that pan-RAF inhibition by LY3009120 potentiates the effect of Ara-C on AML cells, and the combination of these drugs abrogates the protective effect of bone marrow-derived MSC and overcomes MSC-mediated chemoresistance.

We next sought to understand the impact of co-culture with MSCs on cell signaling pathways in OCI-AML3 cells treated for 96 h with LY3009120 and Ara-C alone or in combination. Treatment of OCI/AML3 cells with LY3009120 for 96 h resulted in a remarkable reduction in pERK levels, with or without combination with Ara-C. (Figure 3B) Interestingly, there was a notable reduction in pAKT (Ser473) in OCI/AML3 cells treated with LY3009120 ± Ara-C in the presence of MSCs. This suggests that MSCs might abrogate AKT activation resulting from RAF inhibition under certain conditions. Relative to OCI/AML3 controls, OCI/AML3 cells co-cultured with MSCs had a notable decrease in pERK following exposure to Ara-C.

### 3.4. Concurrent Pan-RAF and FLT3 Inhibition Exerts a Synergistic Antiapoptotic Effect in Sorafenib-Sensitive AML Cell Lines

**Figure 4:**
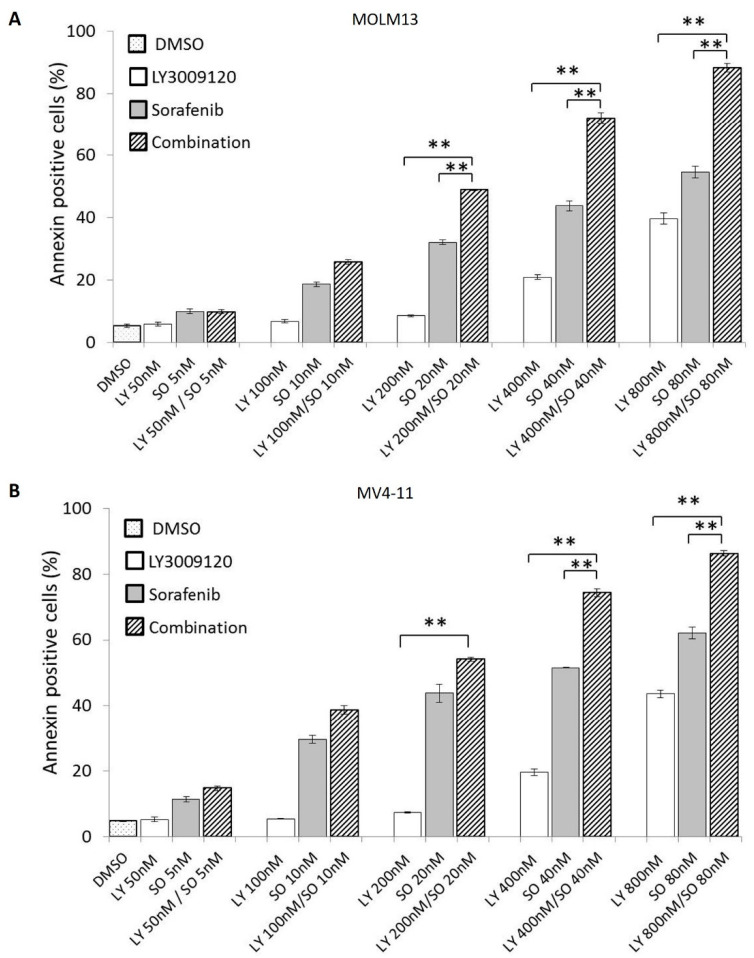
The combination of LY3009120 and sorafenib potentiates their pro-apoptotic effect in *FLT3*-mutated AML cells MOLM13 (**A**) and MV4-11 (**B**). [** *p* < 0.001] [DMSO: dimethyl sulfoxide; LY: LY3009120; SO: sorafenib].

Dual inhibition of FLT3 and the MAPK pathway has demonstrated synergy in vitro and in early-phase clinical trials [7,25]. In view of the limited growth inhibition observed with single-agent LY3009120 in *FLT3*-ITD-mutated AML cell lines, we asked whether the use of a tyrosine kinase inhibitor with FLT3 specificity such as sorafenib might augment the antileukemic effects of LY3009120. Accordingly, we treated MOLM13 and MV4-11 cells with incremental concentrations of LY3009120, sorafenib, and the combination of LY3009120 + sorafenib for 72 h. In each of these cell lines, the LY3009120 + sorafenib combination resulted in significantly higher levels of apoptosis. (Figure 4A,B) The mean CI value for each of the combination doses (LY3009120: 200, 400, 800 nM; sorafenib: 20, 40, 80 nM) was <0.6, indicating positive synergy (Table S2).

## 4. Discussion

Constitutive activation of the MAPK signaling pathway contributes to leukemogenesis, leukemia progression, and chemoresistance [26,27,28,29]. While targeting RAS itself remains elusive in myeloid neoplasms, several inhibitors targeting downstream effectors have been developed in recent years [30]. LY3009120 is a pan-RAF inhibitor with activity against the three RAF isoforms as well as various forms of RAF dimers. Hence, it is capable of inhibiting the phosphorylation of MEK and ERK in different genetic backgrounds and exerts minimal paradoxical pathway activation [20,22,31]. The MAPK pathway is also implicated in stroma-mediated resistance in AML [25]. In this study, we show that pan-RAF inhibition using LY3009120 induces growth inhibition and apoptosis of human AML cells, inhibits MAPK pathway and AKT/mTOR pathway activation, overcomes bone marrow stromal cell-mediated drug resistance to Ara-C, and synergizes with sorafenib to increase apoptosis in AML cells carrying *FLT3* mutations.

Mutational or non-mutational activation of the MAPK pathway can activate collateral pathways, including the AKT/mTOR signaling pathway, and is implicated in acquired resistance to targeted inhibition of FLT3, mutant IDH, and BCL2 relevant to AML therapy. LY3009120 significantly suppressed AKT phosphorylation as well as phosphorylation of the downstream molecules P70S6K and S6K, particularly in OCI-AML3 cells, which carry the *NRAS*^Q61L^ mutation. The authors acknowledge that while data in this study suggest a possible favorable impact of LY3009120 stemming from the presence of *NRAS*^Q61L^ mutation in OCI-AML3, testing a broader set of cell lines or patient samples would be needed before conclusions about the role of underlying genetics in the response of AML cells to LY3009120 can be drawn. Such caution is warranted in view of recent in vitro data showing inconclusive correlations between the response to LY3000120 and the underlying mutational status in AML cell lines or primary AML cells [32]. We also evaluated the level of eIF4E, a downstream target of mTOR that is also regulated by ERK [27]; however, we did not observe changes in eIF4E upon LY3009120 treatment. Given the role of ERK and mTOR in the regulation of apoptosis and proliferation [33,34], our findings suggest that pan-RAF inhibition using LY3009120 results in reduced ERK activation with collateral reduction in AKT/mTOR activation. Although the specific mechanism underlying such effects might be attributed to the RAF-specific inhibitory activity of LY3000120, the inhibitory effect of this molecule on other members of the MAPK signaling pathways is not known. In their original description of LY3009120, Henry et al. [20] showed that while the compound binds to RAF molecules with IC_50_ values ranging from 31–47 nM, other members of the MAPK signaling pathway, namely, MAPK (p38) and MAP3K1, have IC_50_ values of 61–97 nM and 98 nM, respectively. It is not clear from these data whether the binding of LY3009120 to MAPK or MAP3K1 exerts a differential biologic effect that is separate from its binding to RAF molecules, but such a possibility would be postulated to be highly likely.

RPPA data obtained from OCI/AML3 and MV4-11 cells following treatment with LY3009120 demonstrate multiple effects on key cellular functions. This effect of pan-RAF inhibition with LY3009120 appears to be mediated, at least in part, by downstream modulation of cell cycle components such as Wee1, Chk1, PLK1, and cyclin B1, which exhibited the most notable change from the baseline. In addition, there was significant downregulation of S6 in both OCI/AML3 and MV4-11 cells demonstrable in RPPA data and immunoblot assays. Notably, P70S6K was also reduced in MV4-11 cells. The impact of pan-RAF inhibition also impacted JAK/STAT downregulation (Jak2 and STAT3). Tambe et al. demonstrated recently that pan-RAF inhibition results in downregulation of MCL1 and apoptosis of cells that are dependent on MCL1 for survival [32]. We noted such a trend in OCI/AML3 cells but not in MV4-11 cells. Together, these data point to multiple potential synergies that pan-RAF inhibition could exert in the context of targeted therapies for AML, particularly with agents targeting FLT3 and BCL2. These data warrant further exploration in ex vivo models and/or early phase clinical trials.

Ara-C is one of the most effective cytotoxic agents for the treatment of AML and helps achieve high rates of early remission especially in younger patients. Yet, many patients ultimately relapse and succumb to their disease [35], indicating resistance of leukemia cells. The bone marrow stromal microenvironment protects resident leukemic cells and plays a crucial role in AML relapse [36,37]. Activation of the AKT/mTOR and MAPK signaling pathways, upregulation of the anti-apoptotic BCL2 family, and alterations involving the MDM2/TP53 tumor suppressor pathway have been identified in patients with AML relapse in association with stroma-mediated survival advantage for AML cells [38,39,40]. We demonstrate in our study that co-culture of OCI/AML3 cells with bone marrow-derived MSC results in a protective effect during exposure to Ara-C. Notably, LY3009120 seems to help AML cells overcome stroma-mediated resistance, likely via mechanisms that result in a simultaneous reduction in ERK and AKT activation. The apoptosis repressor with caspase recruitment domain (ARC) protein has been shown to play a critical role in conferring drug resistance and survival advantage to AML cells [41,42]. Notably, Mak et al. [41] have shown that MSCs regulate ARC through activation of the MAPK and AKT signaling pathways. As such, it would be intriguing to speculate that LY3009120 abrogates the protective effect of MSCs by secondarily inhibiting ARC.

## 5. Conclusions

In this study, we show that the pan-RAF inhibitor LY3009120 induces apoptosis and inhibits proliferation in AML cells with either *RAS* or *FLT3* mutations. LY3009120 not only targets the RAS/RAF/MEK/ERK pathway but also impacts AKT/mTOR pathway downstream effectors, including p-P70S6K and p-S6. Our data further show that LY3009120 combined with Ara-C overcomes MSC-mediated drug resistance, and the combination of LY3009120 and sorafenib appears to synergistically increase apoptosis in MOLM13 and MV4-11 cells carrying *FLT3* mutations. Together, these pre-clinical studies provide a rationale for continued exploration of safe and effective RAF inhibitors as an adjunct treatment modality for patients with AML.

## Supplementary Materials

The following are available online at https://www.mdpi.com/2072-6694/12/12/3511/s1, Table S1: Reverse protein phase array analysis of AML cells exposed to LY3009120. Table S2: Full dataset of combination treatment of AML cells with LY3009120 and sorafenib.
