# Supplementary material for: Pan-RAF Inhibition Shows Anti-Leukemic Activity in RAS-Mutant Acute Myeloid Leukemia Cells and Potentiates the Effect of Sorafenib in Cells with FLT3 Mutation
Source: Cancers (Basel). 2020 Nov 25;12(12):3511. doi: 10.3390/cancers12123511 (PMC7761301; doi:10.3390/cancers12123511)
Supplement: Supplementary file 1 [file cancers-12-03511-s001.pdf]

TABLE S1

	X1.OCI.0	X2.OCI.24h.LY100nm.	X3.OCI.48h.LY100nm.	X4.MV4.0	X5.MV4.24h.LY500nm.	X6.MV4.48h.LY500nm.	log2FC.OCI.24hVs0h	log2FC.OCI.48hVs0h	log2FC.OCI.48hVs24h	log2FC.MV4.24hVs0h	log2FC.MV4.48hVs0h	log2FC.MV4.48hVs24h
14-3-3-beta-R-V	1.128027329	1.032905245	1.047113724	0.563004238	0.377181257	0.168504712	-0.095122084	-0.080913605	0.014208479	-0.185822981	-0.394499526	-0.208676545
14-3-3-epsilon-M-C	0.34886135	0.251423698	0.295920859	-0.275092949	-0.293380536	-0.56458274	-0.097437652	-0.052940491	0.044497161	-0.018287587	-0.289489791	-0.271202204
14-3-3-zeta-R-V	1.820418438	1.748841403	1.819517309	1.580096861	1.855475683	1.34200381	-0.071577035	-0.000901129	0.070675906	0.275378822	-0.238093051	-0.513471873
4E-BP1-R-V	2.269704368	1.849480725	1.375729146	1.804245357	1.315242782	0.969813987	-0.420223643	-0.893975222	-0.473751579	-0.489002575	-0.83443137	-0.345428795
4E-BP1_pS65-R-V	1.409127532	0.069440323	-0.046537157	0.435247351	0.150081829	-0.047813208	-1.339687209	-1.455664689	-0.11597748	-0.285165522	-0.483060559	-0.197895037
53BP1-R-V	0.673984918	0.558349932	0.176739764	-0.650212398	-0.686808162	-1.085499772	-0.115634986	-0.497245154	-0.381610168	-0.036595764	-0.435287374	-0.39869161
A-Raf-R-V	2.017546893	1.461785959	1.559188217	1.226629284	0.488550339	0.284040669	-0.555760934	-0.458358676	0.097402258	-0.738078945	-0.942588615	-0.20450967
ACC1-R-C	1.167371276	0.450054929	0.33609514	0.033832325	-0.200272508	-1.096179699	-0.717316347	-0.831276136	-0.113959789	-0.234104833	-1.130012024	-0.895907191
ACC_pS79-R-V	0.990057274	0.368145282	-0.062466095	-0.355697619	-0.803584717	-1.360244723	-0.621911992	-1.052523369	-0.430611377	-0.447887098	-1.004547104	-0.556660006
ADAR1-M-V	3.410458691	2.981286322	3.038160017	1.013691228	0.995834332	0.408983579	-0.429172369	-0.372298674	0.056873695	-0.017856896	-0.604707649	-0.586850753
Akt-R-V	3.335108904	2.700746594	2.57477299	1.562295432	1.427491637	0.366039989	-0.63436231	-0.760335914	-0.125973604	-0.134803795	-1.196255443	-1.061451648
Akt-M-S	0.019148564	-0.955563058	-0.974162662	-1.418400054	-1.518340183	-2.05694519	-0.974711622	-0.993311226	-0.018599604	-0.099940129	-0.638545136	-0.538605007
Akt_pS473-R-V	0.177888597	-0.29600764	-0.31000629	-1.351499449	0.420134361	-0.378311641	-0.473896237	-0.487894887	-0.01399865	1.77163381	0.973187808	-0.798446002
Akt_pT308-R-V	0.38609474	0.261721741	0.31716276	-0.246969418	0.41621037	-0.093822879	-0.124372999	-0.06893198	0.055441019	0.663179788	0.153146539	-0.510033249
AMPK-a2_pS345-R-V	-0.484946005	-0.647235575	-0.686472246	-1.261995811	-1.401968491	-1.689379078	-0.16228957	-0.201526241	-0.039236671	-0.13997268	-0.427383267	-0.287410587
AMPKa-R-C	-0.523911989	-0.700859565	-0.7918236	-1.358131636	-1.44688834	-1.579197199	-0.176947576	-0.267911611	-0.090964035	-0.088756704	-0.221065563	-0.132308859
AMPKa_pT172-R-C	2.888999523	2.276779545	2.282481487	0.761231464	0.669304638	0.176647555	-0.612219978	-0.606518036	0.005701942	-0.091926826	-0.584583909	-0.492657083
Annexin-I-M-V	-1.175909945	-1.108122528	-1.019291194	-1.974930878	-1.866193623	-2.069509085	0.067787417	0.156618751	0.088831334	0.108737255	-0.094578207	-0.203315462
Annexin-VII-M-V	1.949483996	1.814205488	2.061382831	0.968889916	1.024436382	0.459508422	-0.135278508	0.111898835	0.247177343	0.055546466	-0.509381494	-0.56492796
AR-R-V	0.971397289	0.788674049	0.760767945	0.189870015	-0.023371827	-0.456961946	-0.18272324	-0.210629344	-0.027906104	-0.213241842	-0.646831961	-0.433590119
ARID1A-R-C	5.63511586	4.95450113	4.697897321	3.443055168	3.616802367	2.245607144	-0.68061473	-0.937218539	-0.256603809	0.173747199	-1.197448024	-1.371195223
Atg3-R-V	3.267543857	2.49333924	2.401936385	1.478071836	1.387712678	1.011097981	-0.774204617	-0.865607472	-0.091402855	-0.090359158	-0.466973855	-0.376614697
Atg7-R-V	1.385388693	1.006327897	0.923926673	-0.073075913	-0.159276784	-0.820470014	-0.379060796	-0.46146202	-0.082401224	-0.086200871	-0.747394101	-0.66119323
ATM-R-V	3.621751333	3.491379821	3.410395385	2.774443811	2.789252391	2.251046507	-0.130371512	-0.211355948	-0.080984436	0.01480858	-0.523397304	-0.538205884
ATM_pS1981-R-V	-1.367264267	-1.440740394	-1.474739967	-2.142650992	-2.258661664	-2.408559635	-0.073476127	-0.1074757	-0.033999573	-0.116010672	-0.265908643	-0.149897971
ATRX-R-C	2.56783464	1.390936518	1.28109685	0.7999173	0.572626087	0.056234637	-1.176898122	-1.28673779	-0.109839668	-0.227291213	-0.743682663	-0.51639145
ATR_pS428-R-C	0.667513957	0.245087894	0.048109385	0.165330503	-0.108564745	-0.748434184	-0.422426063	-0.619404572	-0.196978509	-0.273895248	-0.913764687	-0.639869439
Aurora-B-R-V	0.062649664	-0.236212157	-0.540485541	-0.342948409	-0.959355407	-1.403599306	-0.298861821	-0.603135205	-0.304273384	-0.616406998	-1.060650897	-0.444243899
Axl-R-V	-1.654031108	-1.451972729	-1.605643351	-1.726587376	-0.811772009	-1.519601649	0.202058379	0.048387757	-0.153670622	0.914815367	0.206985727	-0.70782964
b-Actin-R-C	0.103968979	-0.06177163	-0.067597343	-0.702890199	-0.657022204	-1.010935385	-0.165740609	-0.171566322	-0.005825713	0.045867995	-0.308045186	-0.353913181
b-Catenin-R-V	-2.1077915	-2.377934413	-2.457776202	-3.138308851	-3.322645704	-3.322645704	-0.270142913	-0.349984702	-0.079841789	-0.184336853	-0.184336853	0
b-Catenin_pT41_S45-R-V	0.248215619	0.056936125	0.060876968	-0.45796513	-0.561144395	-0.904247925	-0.191279494	-0.187338651	0.003940843	-0.103179265	-0.446282795	-0.34310353
B-Raf-R-V	-0.034520648	-0.168951258	-0.216767205	-0.86399142	-1.036464402	-1.244765859	-0.13443061	-0.182246557	-0.047815947	-0.172472982	-0.380774439	-0.208301457
B-Raf_pS445-R-V	1.583664335	0.67959357	0.631588855	-0.041243753	-0.648564135	-1.360263314	-0.904070765	-0.95207548	-0.048004715	-0.607320382	-1.319019561	-0.711699179
B7-H4-R-C	-0.49998409	-0.656819991	-0.716143573	-1.149966795	-1.218071342	-1.446341935	-0.156835901	-0.216159483	-0.059323582	-0.068104547	-0.29637514	-0.228270593
Bad_pS112-R-V	0.097029371	-0.231081203	-0.326806471	-0.742738969	-0.79805983	-1.213730062	-0.328110574	-0.423835842	-0.095725268	-0.055320861	-0.470991093	-0.415670232
Bak-R-C	-0.462174675	-0.584571955	-0.627036986	-1.331984676	-1.408331961	-1.586784005	-0.12239728	-0.164862311	-0.042465031	-0.076347285	-0.254799329	-0.178452044
BAP1-M-V	0.094849062	-0.041873846	-0.015188799	-0.619895717	-0.689396028	-0.909343344	-0.136722908	-0.110037861	0.026685047	-0.069500311	-0.289447627	-0.219947316
Bax-R-V	-0.144908325	-0.264653016	-0.029704538	0.592687813	0.489712273	0.217182489	-0.119744691	0.115203787	0.234948478	-0.10297554	-0.375505324	-0.272529784
Bcl-xL-R-V	-0.130906918	-0.477328736	-0.522636089	-0.707508106	-0.896425824	-1.142294571	-0.346421818	-0.391729171	-0.045307353	-0.188917718	-0.434786465	-0.245868747
Bcl2-M-V	1.784677492	1.411558839	1.492651585	1.378523388	1.360920135	0.918742684	-0.373118653	-0.292025907	0.081092746	-0.017603253	-0.459780704	-0.442177451
Bcl2A1-R-V	1.118770748	0.938058271	0.962613726	0.189896717	0.175592525	-0.375262703	-0.180712477	-0.156157022	0.024555455	-0.014304192	-0.56515942	-0.550855228
Beclin-G-C	-0.529483904	-0.655937252	-0.669760978	-1.229699061	-1.224909948	-1.570508062	-0.126453348	-0.140277074	-0.013823726	0.004789113	-0.340809001	-0.345598114
Bid-R-C	-0.391617048	-0.595755045	-0.552145817	-0.957722697	-1.068050895	-1.293852098	-0.204137997	-0.160528769	0.043609228	-0.110328198	-0.336129401	-0.225801203
Bim-R-V	0.398178413	0.326218868	0.411988053	-2.093414204	-2.003516618	-2.056385794	-0.071959545	0.01380964	0.085769185	0.089897586	0.03702841	-0.052869176
BMX-R-S	-1.355402114	-1.440810109	-1.405532595	-1.963238836	-2.112930033	-2.331735428	-0.085407995	-0.050130481	0.035277514	-0.149691197	-0.368496592	-0.218805395
BRD4-R-V	4.540976019	4.061483168	3.794800151	2.718235406	2.860323365	1.561948426	-0.479492851	-0.746175868	-0.266683017	0.142087959	-1.15628698	-1.298374939
c-Abl-R-V	1.742982373	1.22560741	1.172730224	0.523348088	0.479216742	-0.087027334	-0.517374963	-0.570252149	-0.052877186	-0.044131346	-0.610375422	-0.566244076
c-IAP2-R-C	-2.053939996	-2.077844822	-2.128874904	-2.620416528	-2.712351147	-2.927244819	-0.023904826	-0.074934908	-0.051030082	-0.091934619	-0.306828291	-0.214893672
c-Jun_pS73-R-V	-1.089914461	-1.340289742	-1.612515737	-1.879892025	-2.182636003	-2.6162627	-0.250375281	-0.522601276	-0.272225995	-0.302743978	-0.736370675	-0.433626697
c-Kit-R-V	-1.312936631	-1.453234603	-1.452445692	-1.429885716	-1.569043026	-1.794598405	-0.140297972	-0.139509061	0.000788911	-0.13915731	-0.364712689	-0.225555379
c-Met-M-Q	2.092382302	1.385168541	1.385324616	0.953284542	0.904017239	0.369365733	-0.707213761	-0.707057686	0.000156075	-0.049267303	-0.583918809	-0.534651506
c-Met_pY1234_Y1235-R-V	1.20926798	1.045536267	1.039883958	0.462353713	0.343788327	-0.024535435	-0.163731713	-0.169384022	-0.005652309	-0.118565386	-0.486889148	-0.368323762
c-Myc-R-C	1.802646228	0.965277406	1.013539791	0.12529031	-0.121973406	-0.767230049	-0.837368822	-0.789106437	0.048262385	-0.247263716	-0.892520359	-0.645256643
C-Raf-R-C	2.877931885	2.375180272	1.94925091	0.804594243	0.792401589	0.155277121	-0.502751613	-0.928680975	-0.425929362	-0.012192654	-0.649317122	-0.637124468
C-Raf_pS338-R-V	1.223789308	0.627137845	0.557409466	-0.612025794	-0.482431048	-0.778863946	-0.596651463	-0.666379842	-0.069728379	0.129594746	-0.166838152	-0.296432898
Caspase-3-R-C	-0.735702577	-0.081144592	-0.527236758	-1.144279352	-1.01460537	-0.807044805	0.654557985	0.208465819	-0.446092166	0.129673982	0.337234547	0.207560565
Caspase-7-cleaved-R-C	-2.408506415	-0.823818425	-1.174585869	-2.869933668	-2.861158033	-2.815903375	1.58468799	1.233920546	-0.350767444	0.008775635	0.054030293	0.045254658
Caspase-8-M-Q	2.718478649	1.95184029	1.929457641	1.270695498	1.252170408	0.623152682	-0.766638359	-0.789021008	-0.022382649	-0.01852509	-0.647542816	-0.629017726
Caveolin-1-R-V	-3.693837784	-3.680832299	-3.710478425	-3.710478425	-3.710478425	-3.710478425	0.013005485	-0.016640641	-0.029646126	0	0	0
CD171-M-V	-1.177439309	-1.363617094	-1.351444229	-1.600115704	-1.624148729	-2.019262814	-0.186177785	-0.17400492	0.012172865	-0.024033025	-0.41914711	-0.395114085
CD26-R-V	0.40191007	0.155161133	0.171275573	-0.331156873	-0.430307081	-0.935230847	-0.246748937	-0.230634497	0.01611444	-0.099150208	-0.604073974	-0.504923766
CD29-M-V	-0.305434762	-0.46837459	-0.47532284	-0.853493335	-0.984484163	-1.223817708	-0.162939828	-0.169888078	-0.00694825	-0.130990828	-0.370324373	-0.239333545
CD31-M-V	-1.456088624	-1.539022737	-1.42254621	-1.738249051	-1.454634337	-2.072744034	-0.082934113	0.033542414	0.116476527	0.283614714	-0.334494983	-0.618109697
CD44-M-C	-0.7265196	-0.883031013	-0.891745262	-1.372826574	-1.500433706	-1.746892885	-0.156511413	-0.165225662	-0.008714249	-0.127607132	-0.374066311	-0.246459179
CD49b-M-V	0.351057085	0.219333559	0.194637121	-0.21118163	-0.387887459	-0.661790594	-0.131723526	-0.156419964	-0.024696438	-0.176705829	-0.450608964	-0.273903135
cdc25C-R-V	0.573861146	0.025066431	-0.543220849	-0.216825806	-0.670036859	-1.413714758	-0.548794715	-1.117081995	-0.56828728	-0.453211053	-1.196888952	-0.743677899
Cdc2_pY15-R-C	0.781984398	-0.199573989	-0.593568402	-0.432994536	-0.956841243	-1.462074541	-0.981558387	-1.3755528	-0.393994413	-0.523846707	-1.029080005	-0.505233298
CDK1-R-C	3.870006307	2.724315187	1.539430822	2.209783505	1.001509677	-0.252307255	-1.14569112	-2.330575485	-1.184884365	-1.208273828	-2.46209076	-1.253816932
Chk1-M-C	3.19764073	1.70818141	0.721982783	1.689609554	0.494534559	-0.515777135	-1.48945932	-2.475657947	-0.986198627	-1.195074995	-2.205386689	-1.010311694
Chk1_pS296-R-V	0.053634985	-0.740723715	-0.823729166	-0.671620404	-1.293058526	-1.638623878	-0.7943587	-0.877364151	-0.083005451	-0.621438122	-0.967003474	-0.345565352
Chk2-M-V	0.675114106	0.285288822	0.229247952	-0.033642194	-0.213969481	-0.837955363	-0.389825284	-0.445866154	-0.05604087	-0.180327287	-0.804313169	-0.623985882
Chk2_pT68-R-C	-1.227875808	-1.295602752	-1.298832065	-1.576847396	-1.64909677	-2.011493765	-0.067726944	-0.070956257	-0.003229313	-0.072249374	-0.434646369	-0.362396995
Claudin-7-R-V	-0.820327328	-0.896049689	-0.992518841	-1.084029302	-1.502229267	-1.644836621	-0.075722361	-0.172191513	-0.096469152	-0.418199965	-0.560807319	-0.142607354
COG3-R-V	0.30448806	0.177628586	-0.176465436	-1.209663349	-1.269414081	-2.053370893	-0.126859474	-0.480953496	-0.354094022	-0.059750732	-0.843707544	-0.783956812
Collagen-VI-R-V	1.968933645	1.877910719	2.127859657	1.990939885	2.022154518	1.514685504	-0.091022926	0.158926012	0.249948938	0.031214633	-0.476254381	-0.507469014
Connexin-43-R-C	-2.802232911	-3.147517842	-3.10798031	-3.791445567	-3.810696396	-4.057200976	-0.345284931	-0.305747399	0.039537532	-0.019250829	-0.265755409	-0.24650458
Cox-IV-R-V	3.709225569	3.532487843	3.651253526	2.734812764	3.604305717	2.857547847	-0.176737726	-0.057972043	0.118765683	0.869492953	0.122735083	-0.74675787
Cox2-R-C	-0.143502968	-0.425507495	-0.453672644	-1.090129689	-1.247929685	-1.570516165	-0.282004527	-0.310169676	-0.028165149	-0.157799996	-0.480386476	-0.32258648
Creb-R-C	-3.410391965	-3.579614835	-3.609409576	-4.083782315	-4.255064604	-4.555390011	-0.16922287	-0.199017611	-0.029794741	-0.171282289	-0.471607696	-0.300325407
Cyclin-B1-R-V	1.718696484	0.073885833	-0.701704133	-0.271456994	-1.617293479	-2.726318908	-1.644810651	-2.420400617	-0.775589966	-1.345836485	-2.454861914	-1.109025429

Cyclin-D1-R-V	0.671583722	0.484276681	0.418001055	-0.163481249	-0.184724697	-0.698345434	-0.187307041	-0.253582667	-0.066275626	-0.021243448	-0.534864185	-0.513620737
Cyclin-D3-M-V	1.169526163	-0.257802456	-0.084912285	-0.544450608	-0.804793566	-1.219229398	-1.427328619	-1.254438448	0.172890171	-0.260342958	-0.67477879	-0.414435832
Cyclin-E1-M-V	1.40856876	0.887801914	0.823203006	0.630589907	0.518521846	0.025916176	-0.520766846	-0.585365754	-0.064598908	-0.112068061	-0.604673731	-0.49260567
Cyclophilin-F-M-V	6.541600714	5.437202614	5.957878603	5.034825267	5.167635638	4.620656078	-1.1043981	-0.583722111	0.520675989	0.132810371	-0.414169189	-0.54697956
D-a-Tubulin-R-V	-1.32398002	-1.427489768	-1.40344605	-1.761951301	-1.971785951	-2.23276638	-0.103509748	-0.07946603	0.024043718	-0.20983465	-0.470815079	-0.260980429
DJ1-R-V	2.443440287	2.20195492	2.218688552	1.324816686	1.312857823	0.657118652	-0.241485367	-0.224751735	0.016733632	-0.011958863	-0.667698034	-0.655739171
DM-Histone-H3-R-V	0.976200198	1.314455293	1.091316008	0.215424513	0.336376872	0.373203556	0.338255095	0.11511581	-0.223139285	0.120952359	0.157779043	0.036826684
DM-K9-Histone-H3-R-C	2.06925519	2.237249344	2.41744875	1.211699183	1.221532423	1.227090851	0.167994154	0.34819356	0.180199406	0.00983324	0.015391668	0.005558428
DUSP4-R-V	-0.465606869	-1.256704833	-1.336417541	-1.156756117	-1.76810693	-2.189097999	-0.791097964	-0.870810672	-0.079712708	-0.611350813	-1.032341882	-0.420991069
E-Cadherin-R-V	-2.733894551	-2.790772187	-2.71990995	-3.295401189	-3.474615303	-3.728530543	-0.056877636	0.013984601	0.070862237	-0.179214114	-0.433129354	-0.25391524
E2F1-M-V	-0.916583912	-1.026161888	-1.06951036	-1.994497535	-2.098037028	-2.673379506	-0.109577976	-0.152926448	-0.043348472	-0.103539493	-0.678881971	-0.575342478
eEF2-R-C	6.15855491	5.369460619	4.571320676	3.28073044	3.507578789	1.387236108	-0.789094291	-1.587234234	-0.798139943	0.226848349	-1.893494332	-2.120342681
eEF2K-R-V	1.308012581	0.825959103	0.746939316	1.066124065	1.249857469	0.655975097	-0.482053478	-0.561073265	-0.079019787	0.183733404	-0.410148968	-0.593882372
EGFR-R-V	0.212801259	0.080660681	0.084170477	-0.656628647	-0.765724452	-1.44578008	-0.132140578	-0.128630782	0.003509796	-0.109095805	-0.789151433	-0.680055628
EGFR_pY1173-R-V	1.08024812	0.880384941	0.871145066	0.426918673	0.330709745	-0.097163234	-0.199863179	-0.209103054	-0.009239875	-0.096208928	-0.524081907	-0.427872979
eIF4E-R-V	3.331918689	2.370856648	2.427887603	2.145780577	2.050892509	1.491451077	-0.961062041	-0.904031086	0.057030955	-0.094888068	-0.6543295	-0.559441432
eIF4E_pS209-R-V	-0.014257592	-0.502104207	-0.517747314	-0.618356401	-1.024855784	-1.29055711	-0.487846615	-0.503489722	-0.015643107	-0.406499383	-0.672200709	-0.265701326
eIF4G-R-C	4.403708241	3.283436172	2.879730855	-1.050397774	-1.193271459	-1.579768395	-1.120272069	-1.523977386	-0.403705317	-0.142873685	-0.529370621	-0.386496936
Elk1_pS383-R-C	1.443269648	1.098317156	1.027934943	0.476159291	0.383802929	-0.249308937	-0.344952492	-0.415334705	-0.070382213	-0.092356362	-0.725468228	-0.633111866
ENY2-M-C	0.46273422	0.202384787	0.309204336	0.368741534	0.236548665	-0.13526605	-0.260349433	-0.153529884	0.106819549	-0.132192869	-0.504007584	-0.371814715
ER-R-V	-1.02757558	-1.099088855	-1.054726435	-1.51885245	-1.626231179	-1.9116704	-0.071513275	-0.027150855	0.04436242	-0.107378729	-0.39281795	-0.285439221
ER-a_pS118-R-V	0.508357477	0.445323849	0.40824798	-0.250466735	-0.355753382	-0.63522477	-0.063033628	-0.100109497	-0.037075869	-0.105286647	-0.384758035	-0.279471388
ERCC1-M-V	1.813864814	1.146363406	1.148139943	0.698372366	0.46919859	0.033206068	-0.667501408	-0.665724871	0.001776537	-0.229173776	-0.665166298	-0.435992522
ERCC5-R-C	3.262420415	2.904411086	2.73136569	0.872328184	0.891106772	0.253295286	-0.358009329	-0.531054725	-0.173045396	0.018778626	-0.61903286	-0.637811486
Ets-1-R-V	1.870137213	0.978857688	1.076072857	0.489073451	0.441362791	-0.262182371	-0.891279525	-0.794064356	0.097215169	-0.04771066	-0.751255822	-0.703545162
FAK-R-C	-2.912212069	-3.056383371	-3.177463746	-3.353582147	-3.536879242	-4.172365376	-0.144171302	-0.265251677	-0.121080375	-0.183297095	-0.818783229	-0.635486134
FAK_pY397-R-V	-2.229982526	-2.24595478	-2.283314205	-2.844197343	-2.918192487	-3.644633096	-0.015972254	-0.053331679	-0.037359425	-0.073995144	-0.800435753	-0.726440609
FASN-R-V	3.512946245	2.565349171	1.95823538	1.234872921	0.778694391	-0.998247362	-0.947597074	-1.554710865	-0.607113791	-0.45617853	-2.233120283	-1.776941753
Fibronectin-R-V	-1.382196975	-1.536795282	-1.400458061	-2.231637649	-2.329180345	-2.415977492	-0.154598307	-0.018261086	0.136337221	-0.097542696	-0.184339843	-0.086797147
FoxM1-R-V	1.7671235	0.799814376	-0.051040104	-0.563472939	-1.305938309	-2.070274113	-0.967309124	-1.818163604	-0.85085448	-0.74246537	-1.506801174	-0.764335804
FoxO3a-R-C	-1.924791115	-2.022984846	-2.013207345	-2.828510306	-2.861453738	-3.195477359	-0.098193731	-0.08841623	0.009777501	-0.032943432	-0.366967053	-0.334023621
FoxO3a_pS318_S321-R-C	-0.69976555	-0.932317392	-1.050732453	-1.501282389	-1.66610105	-1.840173335	-0.232551842	-0.350966903	-0.118415061	-0.164818661	-0.338890946	-0.174072285
FRA-1-R-C	1.872202467	1.157019834	1.147511005	0.34531728	0.300775278	-0.214083778	-0.715182633	-0.724691462	-0.009508829	-0.044542002	-0.559401058	-0.514859056
G6PD-R-V	-1.361938462	-1.409159496	-1.413214572	-1.461693861	-1.500271471	-1.729263501	-0.047221034	-0.05127611	-0.004055076	-0.03857761	-0.26756964	-0.22899203
Gab2-R-V	-0.9040379	-0.686039649	-0.442659573	-0.402650072	-0.387288672	-0.891573979	0.217998251	0.461378327	0.243380076	0.0153614	-0.488923907	-0.504285307
GAPDH-M-C	2.183193053	1.613837202	0.932194153	0.679469787	0.113825365	-1.342240807	-0.569355851	-1.2509989	-0.681643049	-0.565644422	-2.021710594	-1.456066172
GATA3-M-V	-0.180344472	-0.356077763	-0.164891159	-0.724439707	-0.750334468	-0.965331135	-0.175733291	0.015453313	0.191186604	-0.025894761	-0.240891428	-0.214996667
GCLM-R-C	0.908914343	0.788548947	0.777810791	0.204635813	0.07359056	-0.163162989	-0.120365396	-0.131103552	-0.010738156	-0.131045253	-0.367798802	-0.236753549
GCN5L2-R-V	2.244836615	1.522202117	1.418082931	0.839457644	0.734904529	0.372800109	-0.722634498	-0.826753684	-0.104119186	-0.104553115	-0.466657535	-0.36210442
Glutamate-D1-2-R-C	-0.333809426	-0.470472601	-0.322957412	-0.757982692	-0.870657077	-1.144258113	-0.136663175	0.010852014	0.147515189	-0.112674385	-0.386275421	-0.273601036
Glutaminase-R-C	1.311552393	0.942721905	0.870546804	0.064648907	-0.056456563	-0.736545487	-0.368830488	-0.441005589	-0.072175101	-0.12110547	-0.801194394	-0.680088924
Granzyme-B-R-V	1.121019136	0.292177945	0.318869316	-0.282838724	-0.392571955	-0.764056394	-0.828841191	-0.80214982	0.026691371	-0.109733231	-0.48121767	-0.371484439
GSK-3a-b-M-V	3.170788621	2.156030836	2.081952704	1.957231096	1.865134286	1.267440224	-1.014757785	-1.088835917	-0.074078132	-0.09209681	-0.689790872	-0.597694062
GSK-3a-b_pS21_S9-R-V	0.752589094	-0.028843792	-0.229595741	-0.701511152	-0.541111697	-2.258862145	-0.781432886	-0.982184835	-0.200751949	0.160399455	-1.557350993	-1.717750448
Gys-R-V	3.794945094	3.020519709	2.966925244	2.904755663	2.901908079	2.044543567	-0.774425385	-0.82801985	-0.053594465	-0.002847584	-0.860212096	-0.857364512
Gys_pS641-R-V	3.710705834	2.851754798	2.630254185	1.922571644	1.956986124	1.291725377	-0.858951036	-1.080451649	-0.221500613	0.03441448	-0.630846267	-0.665260747
H2AX_pS140-M-C	-0.803551525	1.044055418	0.984508857	-0.740689616	-0.695554246	0.576782547	1.847606943	1.788060382	-0.059546561	0.04513537	1.317472163	1.272336793
HER2-M-V	-0.149606999	-0.261144305	-0.26470878	-0.818057869	-0.938146027	-1.091301871	-0.111537306	-0.115101781	-0.003564475	-0.120088158	-0.273244002	-0.153155844
HER2_pY1248-R-C	-2.421599625	-2.453129879	-2.462462684	-2.948445395	-2.990529319	-3.275378201	-0.031530254	-0.040863059	-0.009332805	-0.042083924	-0.326932806	-0.284848882
HER3-R-V	0.473156705	0.21672525	0.120386669	-0.472928876	-0.537634522	-0.796615473	-0.256431455	-0.352770036	-0.096338581	-0.064705646	-0.323686597	-0.258980951
HER3_pY1289-R-C	1.165726761	0.762930619	0.798723984	0.055203087	-0.013143037	-0.496379712	-0.402796142	-0.367002777	0.035793365	-0.068346124	-0.551582799	-0.483236675
Heregulin-R-V	0.455928721	0.263267711	0.268468076	-0.374194016	-0.417638047	-0.871512663	-0.19266101	-0.187460645	0.005200365	-0.043444031	-0.497318647	-0.453874616
HES1-R-V	-1.121730281	-1.212877008	-1.242374706	-1.951480307	-2.051756255	-2.816980974	-0.091146727	-0.120644425	-0.029497698	-0.100275948	-0.865500667	-0.765224719
Hexokinase-II-R-V	0.002696354	-0.276137366	-0.284692565	-0.738188851	-0.913539024	-1.409703707	-0.27883372	-0.287388919	-0.008555199	-0.175350173	-0.671514856	-0.496164683
Hif-1-alpha-M-C	-1.180030063	-1.382519337	-1.385204473	-1.841331183	-1.893328226	-2.144220582	-0.202489274	-0.20517441	-0.002685136	-0.051997043	-0.302889399	-0.250892356
Histone-H3-R-V	0.31252626	2.216536898	2.803564208	0.139699017	0.647063026	1.458097959	1.904010638	2.491037948	0.58702731	0.507364009	1.318398942	0.811034933
HMGB1-M-N	4.204433229	3.352541792	3.275749504	2.505275886	2.395130072	1.636069956	-0.851891437	-0.928683725	-0.076792288	-0.110145814	-0.86920593	-0.759060116
HSP27-M-C	-2.207630655	-2.41001053	-2.461387476	-3.016016552	-2.945670717	-3.134346524	-0.202379875	-0.253756821	-0.051376946	0.070345835	-0.118329972	-0.188675807
HSP27-M-N	0.118116002	-0.316820646	-0.389061715	-1.138950065	-1.258544136	-2.001794707	-0.434936648	-0.507177717	-0.072241069	-0.119594071	-0.862844642	-0.743250571
HSP27_pS82-R-V	-0.695288171	-1.146118342	-1.580798717	-1.720784794	-1.809284373	-2.080200215	-0.450830171	-0.885510546	-0.434680375	-0.088499579	-0.359415421	-0.270915842
HSP70-R-C	-0.182438922	-0.486406797	-0.629613643	-0.812958792	-1.030892047	-1.315270252	-0.303967875	-0.447174721	-0.143206846	-0.217933255	-0.50231146	-0.284378205
IGF1R_pY1135_Y1136-R-V	0.668310768	0.529397308	0.50026324	-0.017336871	-0.09171335	-0.388631822	-0.13891346	-0.168047528	-0.029134068	-0.074376479	-0.371294951	-0.296918472
IGFBP2-R-V	-2.857252941	-3.223284726	-3.178156143	-1.203250119	-2.075162839	-2.79271555	-0.366031785	-0.320903202	0.045128583	-0.87191272	-1.589465431	-0.717552711
IGFRb-R-C	-0.376112153	-0.623854073	-0.520301917	-0.52173621	-0.864473291	-1.614680226	-0.24774192	-0.144189764	0.103552156	-0.342737081	-1.092944016	-0.750206935
INPP4b-R-V	-0.402461204	-0.657709422	-0.740710136	-1.434798491	-1.511978043	-1.782902351	-0.255248218	-0.338248932	-0.083000714	-0.077179552	-0.34810386	-0.270924308
IR-b-R-C	1.977866494	1.713011738	1.883596872	0.336432816	0.471259797	-0.009451934	-0.264854756	-0.094269622	0.170585134	0.134826981	-0.34588475	-0.480711731
IRF-1-R-C	1.008839765	1.014472255	0.778414178	0.050734088	0.028787	-0.464919318	0.00563249	-0.230425587	-0.236058077	-0.021947088	-0.515653406	-0.493706318
IRS1-R-V	-0.212323301	-0.495116014	-0.576839172	-1.181989998	-1.284910174	-1.618459363	-0.282792713	-0.364515871	-0.081723158	-0.102920176	-0.436469365	-0.333549189
Jagged1-R-V	0.3231057	0.157551882	0.239141836	-0.429952727	-0.508947297	-0.971678568	-0.165553818	-0.083963864	0.081589954	-0.07899457	-0.541725841	-0.462731271
Jak2-R-V	3.977708905	3.249717516	3.135282823	2.189846732	2.046558564	1.235746339	-0.727991389	-0.842426082	-0.114434693	-0.143288168	-0.954100393	-0.810812225
JNK2-R-C	-1.44900424	-1.64529146	-1.746429535	-2.444226867	-2.626080703	-2.933429569	-0.19628722	-0.297425295	-0.101138075	-0.181853836	-0.489202702	-0.307348866
JNK_pT183_Y185-R-V	0.885382533	0.70272695	0.694735045	0.262096147	0.128344817	-0.147871321	-0.182655583	-0.190647488	-0.007991905	-0.13375133	-0.409967468	-0.276216138
LC3A-B-R-C	1.476845133	0.633369737	0.810088215	-0.056737585	-0.139378736	-0.468015028	-0.843475396	-0.666756918	0.176718478	-0.082641151	-0.411277443	-0.328636292
Lck-R-V	-0.153476768	-0.452270334	-0.414949874	-1.976263795	-2.332164981	-2.757567757	-0.298793566	-0.261473106	0.03732046	-0.355901186	-0.781303962	-0.425402776
LDHA-R-C	1.807782983	2.624311516	1.581589866	1.252555249	1.282518966	-0.099556782	0.816528533	-0.226193117	-1.04272165	0.029963717	-1.352112031	-1.382075748
LRP6_pS1490-R-V	-1.266037612	-1.425317294	-1.521208821	-2.084962426	-2.188050222	-2.891365444	-0.159279682	-0.255171209	-0.095891527	-0.103087796	-0.806403018	-0.703315222
MAPK_pT202_Y204-R-V	-1.152999953	-1.842062515	-2.062064439	-2.767003358	-2.417641946	-3.156556248	-0.689062562	-0.909064486	-0.220001924	0.349361412	-0.38955289	-0.738914302
Mcl-1-R-V	2.39508413	1.795747014	1.857961397	0.435233326	0.394405164	0.066796943	-0.599337116	-0.537122733	0.062214383	-0.040828162	-0.368436383	-0.327608221
MCT4-R-V	2.288120621	1.71602098	2.111804431	0.886327129	0.752228005	0.235553528	-0.572099641	-0.17631619	0.395783451	-0.134099124	-0.650773601	-0.516674477
MDM2_pS166-R-V	2.769371598	1.775535785	1.742745362	1.108890968	0.679474891	-0.02033605	-0.993835813	-1.026626236	-0.032790423	-0.429416077	-1.129227018	-0.699810941
MEK1-R-V	1.693066976	1.747431472	1.768102729	1.154324394	1.10003023	0.439680662	0.054364496	0.075035753	0.020671257	-0.054294164	-0.714643732	-0.660349568
MEK1_pS217_S221-R-V	2.580875361	1.65949498	1.644841099	1.02259793	0.938578855	0.287987371	-0.921380381	-0.936034262	-0.014653881	-0.084019075	-0.734610559	-0.650591484
MERIT40_pS29-R-V	-0.865049751	-1.269457803	-1.220456827	-1.412005281	-1.266244487	-1.611723088	-0.404408052	-0.355407076	0.049000976	0.145760794	-0.199717807	-0.345478601

Merlin-R-C	1.141559349	0.590059803	0.597469379	0.314729831	0.166982872	-0.403848498	-0.551499546	-0.54408997	0.007409576	-0.147746959	-0.718578329	-0.57083137
MIF-R-C	1.063542559	0.566357902	0.69798522	0.392780485	0.362546188	-0.119374826	-0.497184657	-0.365557339	0.131627318	-0.030234297	-0.512155311	-0.481921014
MIG6-M-V	2.202017605	1.731550907	1.724401507	1.328716382	1.185233062	0.661857664	-0.470466698	-0.477616098	-0.0071494	-0.14348332	-0.666858718	-0.523375398
MMP2-R-V	-1.243742104	-1.306743074	-1.366273876	-2.228859894	-2.296698122	-2.502105505	-0.06300097	-0.122531772	-0.059530802	-0.067838228	-0.273245611	-0.205407383
Mnk1-R-V	0.986199698	0.189176047	0.24840731	0.218290515	-0.027564628	-0.675009273	-0.797023651	-0.737792388	0.059231263	-0.245855143	-0.893299788	-0.647444645
MSH6-R-C	1.107112254	0.778441356	0.626798236	0.417940766	0.157317706	-0.545082085	-0.328670898	-0.480314018	-0.15164312	-0.26062306	-0.963022851	-0.702399791
MSI2-R-C	-0.307473667	-0.384078647	-0.438841811	-1.04140338	-1.054712975	-1.692115818	-0.07660498	-0.131368144	-0.054763164	-0.013309595	-0.650712438	-0.637402843
mTOR-R-V	5.571972874	4.678235357	3.773076548	2.013825824	2.039438853	0.976368721	-0.893737517	-1.798896326	-0.905158809	0.025613029	-1.037457103	-1.063070132
mTOR_pS2448-R-C	2.11724008	0.981439543	1.062207297	0.442194917	0.275329892	0.135810874	-1.135800537	-1.055032783	0.080767754	-0.166865025	-0.306384043	-0.139519018
Myosin-11-R-V	-0.679702452	-0.793166156	-0.946487871	-1.214430838	-1.379252024	-1.621267631	-0.113463704	-0.266785419	-0.153321715	-0.164821186	-0.406836793	-0.242015607
Myosin-IIa_pS1943-R-V	4.441026894	3.873047297	3.75672364	3.34619172	3.363435333	1.71505453	-0.567979597	-0.684303254	-0.116323657	0.017243613	-1.63113719	-1.648380803
Myt1-R-C	1.144203585	0.386306332	0.113149331	0.398074841	-0.114901411	-0.641217943	-0.757897253	-1.031054254	-0.273157001	-0.512976252	-1.039292784	-0.526316532
N-Cadherin-R-V	0.075095924	-0.087071382	-0.085911425	-0.591643635	-0.695776566	-1.083099085	-0.162167306	-0.161007349	0.001159957	-0.104132931	-0.49145545	-0.387322519
N-Ras-M-V	0.009495115	-0.044199617	-0.010646391	-0.772060754	-0.811966236	-0.973244611	-0.053694732	-0.020141506	0.033553226	-0.039905482	-0.201183857	-0.161278375
NAPSIN-A-R-C	2.212719169	1.468279213	1.444501499	0.805292415	0.668078141	0.061098765	-0.744439956	-0.76821767	-0.023777714	-0.137214274	-0.74419365	-0.606979376
NDRG1_pT346-R-V	1.950026607	0.824815268	0.263770001	0.101202131	-0.183870671	-0.564548396	-1.125211339	-1.686256606	-0.561045267	-0.285072802	-0.665750527	-0.380677725
NDUFB4-M-V	-0.1883711	-0.281659035	-0.225506362	-0.762317596	-0.847445191	-1.058285092	-0.093287935	-0.037135262	0.056152673	-0.085127595	-0.295967496	-0.210839901
NF-kB-p65_pS536-R-C	-0.841997738	-1.010520662	-1.086339508	-1.895465615	-2.022052941	-2.196720096	-0.168522924	-0.24434177	-0.075818846	-0.126587326	-0.301254481	-0.174667155
Notch1-R-V	3.501542964	2.388181789	2.550359992	0.805520113	0.64223073	0.006190865	-1.113361175	-0.951182972	0.162178203	-0.163289383	-0.799329248	-0.636039865
Notch3-R-C	1.698777117	1.48197878	1.581890826	0.626927403	0.379833773	-0.237983049	-0.216798337	-0.116886291	0.099912046	-0.24709363	-0.864910452	-0.617816822
Oct-4-R-C	-0.224465837	-0.401922152	-0.453665224	-0.977704186	-1.119465657	-2.06358447	-0.177456315	-0.229199387	-0.051743072	-0.141761471	-1.085880284	-0.944118813
P-Cadherin-R-C	0.705694193	0.577436004	0.707987867	0.087498817	0.048976315	-0.542641855	-0.128258189	0.002293674	0.130551863	-0.038522502	-0.630140672	-0.59161817
p16INK4a-R-V	-0.095635348	-0.63531736	-0.597090163	-1.93050523	-1.982156524	-2.206143598	-0.539682012	-0.501454815	0.038227197	-0.051651294	-0.275638368	-0.223987074
p21-R-V	0.127210455	0.034860292	0.019667835	-0.663770013	-0.567005892	-0.676472214	-0.092350163	-0.10754262	-0.015192457	0.096764121	-0.012702201	-0.109466322
p27-Kip-1-R-V	-0.303318041	-0.417456271	-0.345407294	-0.908669272	-0.861122394	-1.04056606	-0.11413823	-0.042089253	0.072048977	0.047546878	-0.131896788	-0.179443666
p27_pT198-R-V	0.925280093	0.699366745	0.629547762	0.20444835	0.080378819	-0.303070345	-0.225913348	-0.295732331	-0.069818983	-0.124069531	-0.507518695	-0.383449164
p38-MAPK-R-V	5.903665535	5.165319631	4.727527759	3.851639349	3.849365981	2.851328571	-0.738345904	-1.176137776	-0.437791872	-0.002273368	-1.000310778	-0.99803741
p38_pT180_Y182-R-V	-1.562277589	-4.498419349	-4.564919062	-4.76940208	-5.851522441	-6.529613632	-2.93614176	-3.002641473	-0.066499713	-1.082120361	-1.760211552	-0.678091191
p44-42-MAPK-R-V	2.085954751	1.432118631	1.419032629	0.635974573	0.592678351	-0.010518866	-0.65383612	-0.666922122	-0.013086002	-0.043296222	-0.646493439	-0.603197217
p53-R-C	-0.020036541	-0.213739738	-0.354459107	-0.119444448	-0.493026728	-1.066494368	-0.193703197	-0.334422566	-0.140719369	-0.37358228	-0.94704992	-0.57346764
p70-S6K1-R-V	4.641810654	4.152361194	4.126753654	2.405096357	2.304015133	1.384616526	-0.48944946	-0.515057	-0.02560754	-0.101081224	-1.020479831	-0.919398607
p70-S6K_pT389-R-V	1.96423613	1.055417476	1.079763023	0.464113854	0.235624557	-0.035912499	-0.908818654	-0.884473107	0.024345547	-0.228489297	-0.500026353	-0.271537056
p90RSK_pT573-R-C	1.979111808	0.045138084	-0.233666998	-0.524940961	-0.422386452	-1.526008219	-1.933973724	-2.212778806	-0.278805082	0.102554509	-1.001067258	-1.103621767
PAI-1-M-V	-0.31823738	-0.641341347	-0.597389273	-0.990518349	-1.076986173	-1.483782175	-0.323103967	-0.279151893	0.043952074	-0.086467824	-0.493263826	-0.406796002
PAICS-R-C	2.113702385	1.761687484	1.577984773	0.993595963	0.579592384	0.138723465	-0.352014901	-0.535717612	-0.183702711	-0.414003579	-0.854872498	-0.440868919
PAK1-R-V	1.037252628	0.801005751	0.77539982	-0.450559506	-0.424391249	-0.694451226	-0.236246877	-0.261852808	-0.025605931	0.026168257	-0.24389172	-0.270059977
PAK4-R-V	-0.521847065	-0.714711111	-0.858329211	-1.138849918	-1.207186908	-1.470654351	-0.192864046	-0.336482146	-0.1436181	-0.06833699	-0.331804433	-0.263467443
PAR-R-C	-0.38281034	1.36677254	3.903034007	1.52264078	1.458956947	2.86052966	1.74958288	4.285844347	2.536261467	-0.063683833	1.33788888	1.401572713
PARP1-R-V	4.264953808	3.525195719	3.394464188	2.514293188	2.599116608	1.698982127	-0.739758089	-0.87048962	-0.130731531	0.08482342	-0.815311061	-0.900134481
Paxillin-R-C	-1.081791666	-1.236607627	-1.329372949	-2.076225843	-2.182128853	-2.773035087	-0.154815961	-0.247581283	-0.092765322	-0.10590301	-0.696809244	-0.590906234
PCNA-M-C	0.878761747	0.695480421	0.467820623	0.667327004	0.381407618	-0.188178795	-0.183281326	-0.410941124	-0.227659798	-0.285919386	-0.855505799	-0.569586413
PD-L1-R-C	0.119098214	-0.029950122	-0.02989471	-0.530312101	-0.607687796	-0.892847829	-0.149048336	-0.148992924	5.54E-05	-0.077375695	-0.362535728	-0.285160033
Pdcd4-R-C	3.891979067	3.540306451	3.791628992	3.154249443	3.229072087	2.731480716	-0.351672616	-0.100350075	0.251322541	0.074822644	-0.422768727	-0.497591371
PDGFR-b-R-V	-2.335540477	-2.469763765	-2.479033117	-3.107399455	-3.202951507	-3.548044437	-0.134223288	-0.14349264	-0.009269352	-0.095552052	-0.440644982	-0.34509293
PDHK1-R-C	-0.148396319	-0.250143489	-0.248595662	-0.842916497	-0.93833517	-1.200063919	-0.10174717	-0.100199343	0.001547827	-0.095418673	-0.357147422	-0.261728749
PDK1-R-V	1.06248435	0.802221676	0.777911434	0.38942024	0.368425181	-0.166650958	-0.260262674	-0.284572916	-0.024310242	-0.020995059	-0.556071198	-0.535076139
PDK1_pS241-R-V	2.706174822	2.455497649	2.249708293	1.539105231	1.674064957	0.478707006	-0.250677173	-0.456466529	-0.205789356	0.134959726	-1.060398225	-1.195357951
PEA-15-R-V	2.850306911	1.80752578	1.75189964	1.493212709	1.39782933	0.704688354	-1.042781131	-1.098407271	-0.05562614	-0.095383379	-0.788524355	-0.693140976
PEA-15_pS116-R-V	-0.040153491	-0.239756452	-0.244098532	-0.785722597	-0.85508778	-1.115295926	-0.199602961	-0.203945041	-0.00434208	-0.069365183	-0.329573329	-0.260208146
PI3K-p110-a-R-C	0.511295862	0.211032726	0.068708811	-0.789817848	-0.841199722	-1.56300822	-0.300263136	-0.442587051	-0.142323915	-0.051381874	-0.773190372	-0.721808498
PI3K-p110-b-M-C	1.623494825	1.785780107	1.541577639	0.94001184	0.90590837	0.336629719	0.162285282	-0.081917186	-0.244202468	-0.03410347	-0.603382121	-0.569278651
PI3K-p85-R-V	3.43439528	2.649484766	2.731738446	1.595359876	1.932368309	1.109245009	-0.784910514	-0.702656834	0.08225368	0.337008433	-0.486114867	-0.8231233
PKA-a-R-V	2.908540193	2.558979147	2.262425438	1.943244891	2.203867767	1.270827529	-0.349561046	-0.646114755	-0.296553709	0.260623106	-0.672417362	-0.933040468
PKC-b-II_pS660-R-V	3.197392961	2.503527445	2.18852786	0.497647825	-0.02066743	-0.883488463	-0.693865516	-1.008865101	-0.314999585	-0.518315255	-1.381136288	-0.862821033
PKC-delta_pS664-R-V	-0.426047498	-0.534877529	-0.530578197	-1.011056519	-1.088954042	-1.433502758	-0.108830031	-0.104530699	0.004299332	-0.077897523	-0.422446239	-0.344548716
PKCa-R-V	0.579296561	0.329893129	0.419206199	-0.916589566	-1.248095404	-1.569371646	-0.249403432	-0.160090362	0.08931307	-0.331505838	-0.65278208	-0.321276242
PKM2-R-C	1.97849291	1.410489615	1.327311038	0.212020293	0.211626299	0.107828665	-0.568003295	-0.651181872	-0.083178577	-0.000393994	-0.104191628	-0.103797634
PLC-gamma2_pY759-R-C	-0.085392303	-0.254974334	-0.292350531	-0.563508272	-0.680111656	-0.938923434	-0.169582031	-0.206958228	-0.037376197	-0.116603384	-0.375415162	-0.258811778
PLK1-R-C	3.189273641	1.192125989	0.043076472	2.018710421	0.416009488	-0.795677972	-1.997147652	-3.146197169	-1.149049517	-1.602700933	-2.814388393	-1.21168746
PMS2-R-V	-0.072692854	-0.310104873	-0.568157413	-0.909997946	-0.957841069	-1.502230559	-0.237412019	-0.495464559	-0.25805254	-0.047843123	-0.592232613	-0.54438949
PR-R-V	-2.292700001	-2.361744153	-2.353190042	-2.978656092	-3.120443607	-3.391464134	-0.069044152	-0.060490041	0.008554111	-0.141787515	-0.412808042	-0.271020527
PRAS40-M-C	0.985797051	0.701373512	0.60819286	0.239731839	-0.223264195	-0.855032406	-0.284423539	-0.377604191	-0.093180652	-0.462996034	-1.094764245	-0.631768211
PRAS40_pT246-R-V	3.127839423	2.096716179	2.030352552	1.420785569	1.416302163	0.589352739	-1.031123244	-1.097486871	-0.066363627	-0.004483406	-0.83143283	-0.826949424
PREX1-R-V	2.875417115	2.756069674	2.807751156	1.68092626	1.918764008	1.132249816	-0.119347441	-0.067665959	0.051681482	0.237837748	-0.548676444	-0.786514192
PTEN-R-V	4.388098638	4.011170231	3.998164801	4.04449547	4.336848801	3.736511514	-0.376928407	-0.389933837	-0.01300543	0.292353331	-0.307983956	-0.600337287
Rab11-R-E	0.976573934	0.802523149	0.799838284	0.269352775	0.187047208	-0.278227677	-0.174050785	-0.17673565	-0.002684865	-0.082305567	-0.547580452	-0.465274885
Rab25-R-V	-0.117333268	-0.665219058	-0.731284674	-1.65972534	-1.751235619	-2.052861764	-0.54788579	-0.613951406	-0.066065616	-0.091510279	-0.393136424	-0.301626145
Rad50-M-V	1.149012332	1.0559638	1.124142676	0.567814428	0.463810779	0.157197058	-0.093048532	-0.024869656	0.068178876	-0.104003649	-0.41061737	-0.306613721
Rad51-R-V	-0.92689647	-1.078132223	-1.170789709	-1.733073567	-1.857026466	-2.051033015	-0.151235753	-0.243893239	-0.092657486	-0.123952899	-0.317959448	-0.194006549
Raptor-R-V	1.895421539	1.797619303	1.768485134	0.371220908	0.343507461	-0.432857086	-0.097802236	-0.126936405	-0.029134169	-0.027713447	-0.804077994	-0.776364547
Rb-M-Q	0.096237191	-0.133557546	-0.272505242	-0.459001855	-0.7113045	-0.974447478	-0.229794737	-0.368742433	-0.138947696	-0.252302645	-0.515445623	-0.263142978
RBM15-R-V	1.356870826	1.194640827	1.22219204	0.519974958	0.466229787	-0.030258674	-0.162229999	-0.134678786	0.027551213	-0.053745171	-0.550233632	-0.496488461
Rb_pS807_S811-R-V	3.989722378	1.575984833	0.527123848	3.07880536	1.011838159	-0.124390068	-2.413737545	-3.46259853	-1.048860985	-2.066967201	-3.203195428	-1.136228227
Rheb-M-C	3.142687143	2.246594523	2.230565892	1.495461928	1.339762942	0.766372343	-0.89609262	-0.912121251	-0.016028631	-0.155698986	-0.729089585	-0.573390599
Rictor-R-C	2.781366373	1.971448736	1.624615551	-0.232350097	-0.477580263	-1.152732345	-0.809917637	-1.156750822	-0.346833185	-0.245230166	-0.920382248	-0.675152082
Rictor_pT1135-R-V	1.000962612	0.715181314	0.708824585	0.018376935	-0.089056219	-0.767307604	-0.285781298	-0.292138027	-0.006356729	-0.107433154	-0.785684539	-0.678251385
RIP-R-C	-0.388631049	-0.69841249	-0.889213281	-1.903613088	-1.992948708	-2.779050143	-0.309781441	-0.500582232	-0.190800791	-0.08933562	-0.875437055	-0.786101435
Rock-1-R-C	1.684154768	1.156861283	1.083121701	0.417925715	0.391046999	-0.190826825	-0.527293485	-0.601033067	-0.073739582	-0.026878716	-0.60875254	-0.581873824
RPA32-T-C	0.324742994	0.062030252	0.040392571	-0.261105889	-0.37550652	-0.758328257	-0.262712742	-0.284350423	-0.021637681	-0.114400631	-0.497222368	-0.382821737
RPA32_pS4_S8-R-C	2.795000715	1.998785862	1.958880174	1.085890522	0.521464185	-0.181166388	-0.796214853	-0.836120541	-0.039905688	-0.564426337	-1.26705691	-0.702630573
RSK-R-C	2.693086855	1.814090305	1.642832797	0.602215926	0.500968983	-0.194680004	-0.87899655	-1.050254058	-0.171257508	-0.101246943	-0.79689593	-0.695648987
S6-M-V	4.933081115	4.647552679	4.121173926	3.935533924	3.868620925	2.826523946	-0.285528436	-0.811907189	-0.526378753	-0.066912999	-1.109009978	-1.042096979
S6_pS235_S236-R-V	4.117718387	0.447807082	0.663328895	2.416607629	0.310409112	-1.24697559	-3.669911305	-3.454389492	0.215521813	-2.106198517	-3.663583219	-1.557384702

S6_pS240_S244-R-V	4.199113289	0.04796669	-0.043674901	1.12418395	-0.567496834	-1.40502055	-4.151146599	-4.24278819	-0.091641591	-1.691680784	-2.5292045	-0.837523716
SCD-M-V	-0.713117229	-0.837795698	-0.82616064	-1.332243259	-1.440139922	-1.656556279	-0.124678469	-0.113043411	0.011635058	-0.107896663	-0.32431302	-0.216416357
SDHA-R-V	0.389319067	0.670426193	0.531285812	-0.46077938	-0.626336188	-1.069992552	0.281107126	0.141966745	-0.139140381	-0.165556808	-0.609213172	-0.443656364
SF2-M-V	3.618416996	2.826775987	2.855321379	2.018213273	1.967501308	1.117946997	-0.791641009	-0.763095617	0.028545392	-0.050711965	-0.900266276	-0.849554311
Shc_pY317-R-V	0.508262107	0.286499667	0.29025928	-0.679432859	-0.707619392	-1.192026093	-0.22176244	-0.218002827	0.003759613	-0.028186533	-0.512593234	-0.484406701
SHP-2_pY542-R-C	-0.812227848	-1.063914536	-1.042038467	-0.973944464	-0.420189649	-0.761335089	-0.251686688	-0.229810619	0.021876069	0.553754815	0.212609375	-0.34114544
SLC1A5-R-C	2.36391698	1.501879146	1.372014963	1.909398756	1.29688767	0.617349241	-0.862037834	-0.991902017	-0.129864183	-0.612511086	-1.292049515	-0.679538429
Slfn11-G-C	-0.110180615	-0.61842968	-0.699957613	-1.52662069	-1.646001639	-2.111619257	-0.508249065	-0.589776998	-0.081527933	-0.119380949	-0.584998567	-0.465617618
Smac-M-Q	0.426450756	0.033021758	-0.013622289	-0.039679678	-0.163833152	-0.290750595	-0.393428998	-0.440073045	-0.046644047	-0.124153474	-0.251070917	-0.126917443
Smad1-R-V	-1.048542123	-1.269261828	-1.200315388	-1.508259695	-1.553879021	-1.765324585	-0.220719705	-0.151773265	0.06894644	-0.045619326	-0.25706489	-0.211445564
Smad3-R-V	3.17454652	2.512751622	2.453981165	1.56328042	1.47657437	0.830562732	-0.661794898	-0.720565355	-0.058770457	-0.08670605	-0.732717688	-0.646011638
Smad4-M-V	1.8958689	1.271337685	1.115129846	0.343240879	0.273617441	-0.159468571	-0.624531215	-0.780739054	-0.156207839	-0.069623438	-0.50270945	-0.433086012
Snail-M-Q	1.030348286	-0.558952841	-0.624839918	-1.176205026	-1.384285809	-1.852277374	-1.589301127	-1.655188204	-0.065887077	-0.208080783	-0.676072348	-0.467991565
SOD1-M-V	1.703850192	1.163930846	1.655593653	0.656597526	0.642348813	0.331365552	-0.539919346	-0.048256539	0.491662807	-0.014248713	-0.325231974	-0.310983261
SOD2-R-V	-31.88500873	-32.28840071	-31.90616724	-34.03242812	-34.37163343	-34.25021941	-0.40339198	-0.02115851	0.38223347	-0.33920531	-0.21779129	0.12141402
Sox2-R-V	-1.040387742	-1.108200158	-1.110209797	-1.593910937	-1.717824833	-1.984976456	-0.067812416	-0.069822055	-0.002009639	-0.123913896	-0.391065519	-0.267151623
Src-M-V	0.369769039	0.207566631	0.235014253	-0.46485888	-0.545141574	-0.9287034	-0.162202408	-0.134754786	0.027447622	-0.080282694	-0.46384452	-0.383561826
Src_pY416-R-V	2.232968093	0.563940215	0.589478089	-0.018594462	-0.159387673	-0.75297316	-1.669027878	-1.643490004	0.025537874	-0.140793211	-0.734378698	-0.593585487
Src_pY527-R-V	1.833552985	1.353385461	1.639335755	1.342942873	0.957189072	0.273446278	-0.480167524	-0.19421723	0.285950294	-0.385753801	-1.069496595	-0.683742794
Stat3-R-C	2.7681024	2.088381704	1.723933207	1.232132667	1.329535842	0.032970221	-0.679720696	-1.044169193	-0.364448497	0.097403175	-1.199162446	-1.296565621
Stat3_pY705-R-V	0.288436598	0.032639299	0.009860422	-0.246622365	-0.431016572	-0.776105028	-0.255797299	-0.278576176	-0.022778877	-0.184394207	-0.529482663	-0.345088456
Stat5a-R-V	1.864523654	1.294937977	1.35535803	1.202314943	1.224826732	0.375411432	-0.569585677	-0.509165624	0.060420053	0.022511789	-0.826903511	-0.8494153
Stathmin-1-R-V	1.516473577	0.909106075	0.933895438	0.802139828	0.683342633	0.181122717	-0.607367502	-0.582578139	0.024789363	-0.118797195	-0.621017111	-0.502219916
Syk-M-V	4.121833004	3.205970883	3.304909586	3.111026488	3.084409394	2.355810415	-0.915862121	-0.816923418	0.098938703	-0.026617094	-0.755216073	-0.728598979
Tau-M-C	0.941781244	0.751647929	0.652976463	0.121467935	0.148540302	-0.302409416	-0.190133315	-0.288804781	-0.098671466	0.027072367	-0.423877351	-0.450949718
TAZ-R-V	0.314701799	0.002960898	-0.396579538	-1.692708227	-1.832425948	-2.304311115	-0.311740901	-0.711281337	-0.399540436	-0.139717721	-0.611602888	-0.471885167
TFAM-R-V	3.12495011	2.998617048	3.311370243	2.21438996	2.224324692	1.872100757	-0.126333062	0.186420133	0.312753195	0.009934732	-0.342289203	-0.352223935
TFRC-R-V	1.391884933	0.705446005	0.204124504	1.566912337	1.293043981	0.70432639	-0.686438928	-1.187760429	-0.501321501	-0.273868356	-0.862585947	-0.588717591
TIGAR-R-V	2.359827474	2.003146083	1.996348144	1.560715653	1.494828373	0.610141913	-0.356681391	-0.36347933	-0.006797939	-0.06588728	-0.95057374	-0.88468646
Transglutaminase-M-V	-0.076964242	-0.193664453	-0.208111603	-0.846506691	-0.923925843	-1.508091853	-0.116700211	-0.131147361	-0.01444715	-0.077419152	-0.661585162	-0.58416601
TRIM25-R-C	4.989727856	4.430795702	4.536195695	3.412735663	3.6956949	2.595973846	-0.558932154	-0.453532161	0.105399993	0.282959237	-0.816761817	-1.099721054
TSC1-R-C	2.755861454	2.394044693	1.84840933	0.62602185	0.466429682	-0.547816565	-0.361816761	-0.907452124	-0.545635363	-0.159592168	-1.173838415	-1.014246247
TTF1-R-V	0.440863777	0.254657009	0.040882342	-0.39377463	-0.5775194	-1.078646886	-0.186206768	-0.399981435	-0.213774667	-0.18374477	-0.684872256	-0.501127486
Tuberin-R-V	1.888270351	0.969225937	0.922819808	0.210451612	0.219087817	-0.436927304	-0.919044414	-0.965450543	-0.046406129	0.008636205	-0.647378916	-0.656015121
Tuberin_pT1462-R-V	1.222333009	1.144799735	1.109385521	0.034870466	0.070794894	-0.34516064	-0.077533274	-0.112947488	-0.035414214	0.035924428	-0.380031106	-0.415955534
TUFM-R-V	0.042432436	-0.101467903	-0.118885217	-0.717537978	-0.872643852	-1.127482219	-0.143900339	-0.161317653	-0.017417314	-0.155105874	-0.409944241	-0.254838367
TWIST-M-C	-0.026959994	-0.194980562	-0.197163783	-0.800378206	-0.910692716	-1.130366503	-0.168020568	-0.170203789	-0.002183221	-0.11031451	-0.329988297	-0.219673787
Tyro3-R-V	0.182441067	-0.038513545	-0.142524985	-0.641838278	-0.755444227	-1.305188546	-0.220954612	-0.324966052	-0.10401144	-0.113605949	-0.663350268	-0.549744319
UBAC1-R-V	1.973784672	1.0509751	1.067560089	0.783779187	0.647432866	0.160520032	-0.922809572	-0.906224583	0.016584989	-0.136346321	-0.623259155	-0.486912834
Ubq-Histone-H2B-M-C	-0.368552822	-1.065131223	-1.196838401	-1.760154217	-2.24352076	-3.603923652	-0.696578401	-0.828285579	-0.131707178	-0.483366543	-1.843769435	-1.360402892
UGT1A-M-V	-0.331663206	-0.554187402	-0.567861745	-0.954111913	-1.049309107	-1.365706155	-0.222524196	-0.236198539	-0.013674343	-0.095197194	-0.411594242	-0.316397048
ULK1_pS757-R-C	-0.058484561	-0.668057423	-0.648314177	-1.499989423	-1.549091431	-2.07953254	-0.609572862	-0.589829616	0.019743246	-0.049102008	-0.579543117	-0.530441109
VASP-R-V	1.546127589	0.807671874	0.797518404	1.183487619	0.935520964	0.538657258	-0.738455715	-0.748609185	-0.01015347	-0.247966655	-0.644830361	-0.396863706
VEGFR-2-R-V	2.054604949	1.916640271	1.942831224	0.567058667	0.51850175	-0.065350714	-0.137964678	-0.111773725	0.026190953	-0.048556917	-0.632409381	-0.583852464
VHL-EPPK1-M-E	-0.947616384	-0.786696636	-1.050539676	-1.361528323	-1.534139077	-1.555556546	0.160919748	-0.102923292	-0.26384304	-0.172610754	-0.194028223	-0.021417469
Vimentin-M-C	-1.073084932	-1.116807815	-0.850526376	-1.725311068	-1.747351947	-1.846992878	-0.043722883	0.222558556	0.266281439	-0.022040879	-0.12168181	-0.099640931
Wee1-R-C	1.171437711	0.04375076	-0.2524234	-0.35041735	-1.00537411	-1.473653592	-1.127686951	-1.423861111	-0.29617416	-0.65495676	-1.123236242	-0.468279482
Wee1_pS642-R-C	0.511776238	-0.530217331	-0.881042342	-0.828316706	-1.328673247	-1.467391523	-1.041993569	-1.39281858	-0.350825011	-0.500356541	-0.639074817	-0.138718276
WIPI1-R-C	1.703853834	0.601236693	0.671722535	-0.63121611	-0.633655171	-0.819897055	-1.102617141	-1.032131299	0.070485842	-0.002439061	-0.188680945	-0.186241884
WIPI2-R-C	0.664880014	0.588728691	0.572724219	0.04538704	0.018025333	-0.4874968	-0.076151323	-0.092155795	-0.016004472	-0.027361707	-0.53288384	-0.505522133
XBP-1-G-C	-0.449549822	-1.023034391	-0.988487351	-1.260934279	-1.349670948	-1.831589735	-0.573484569	-0.538937529	0.03454704	-0.088736669	-0.570655456	-0.481918787
XPA-M-V	2.030580516	1.723586858	1.58832926	0.46708312	0.984657911	-0.676688451	-0.306993658	-0.442251256	-0.135257598	0.517574791	-1.143771571	-1.661346362
XPF-R-C	-0.022960161	-0.203152325	-0.222918273	-0.787340056	-0.869503799	-1.07416096	-0.180192164	-0.199958112	-0.019765948	-0.082163743	-0.286820904	-0.204657161
XRCC1-R-C	-0.044163992	-0.192671432	-0.363076595	-0.183710535	-0.348957739	-0.759206517	-0.14850744	-0.318912603	-0.170405163	-0.165247204	-0.575495982	-0.410248778
YAP-R-E	1.637553952	0.943451895	1.510858852	0.839834661	0.890363431	0.570243821	-0.694102057	-0.1266951	0.567406957	0.05052877	-0.26959084	-0.32011961
YAP_pS127-R-E	-1.240140908	-1.318545419	-1.523482898	-2.612090249	-2.87420986	-2.897176379	-0.078404511	-0.28334199	-0.204937479	-0.262119611	-0.28508613	-0.022966519
YB1_pS102-R-V	1.519107415	0.393092215	0.330182749	0.033209596	-0.340693991	-1.05235003	-1.1260152	-1.188924666	-0.062909466	-0.373903587	-1.085559626	-0.711656039
ZAP-70-R-C	-1.198227893	-1.299516078	-1.296863106	-1.817028139	-1.975112686	-2.254456594	-0.101288185	-0.098635213	0.002652972	-0.158084547	-0.437428455	-0.279343908

TABLE S2

**MOLM-13**				
Annexin V positive cells	%	SE	T-test	P value
DMSO	5.3	0.5		
LY 50nM	5.8	0.6		
SO 5nM	9.9	0.740998		
LY 50nM / SO 5nM	9.8	0.584076		
LY 100nM	6.7	0.5		
SO 10nM	18.67667	0.730441		
LY 100nM/SO 10nM	25.67333	0.756865		
LY 200nM	8.5	0.3	3.2E-08	<0.001
SO 20nM	32.03667	0.730441	0.000011	<0.001
LY 200nM/SO 20nM	48.9	0.223681		
LY 400nM	20.8	0.7	0.000011	<0.001
SO 40nM	43.7	1.535676	0.000268	<0.001
LY 400nM/SO 40nM	71.94333	1.7579		
LY 800nM	39.7	1.7	0.000025	
SO 80nM	54.65	1.890079	0.000136	
LY 800nM/SO 80nM	88.2	1.364726		

**MV4-11**				
Annexin V positive cells	%	SE	T-test	P value
DMSO	4.8	0.1		
LY 50nM	5.3	0.7		
SO 5nM	11.45667	0.8		
LY 50nM / SO 5nM	14.73667	0.757679		
LY 100nM	5.4	0.1		
SO 10nM	29.68333	1.25		
LY 100nM/SO 10nM	38.68667	1.3		
LY 200nM	7.4	0.2	1.69E-07	<0.001
SO 20nM	43.80667	2.7	0.021686	
LY 200nM/SO 20nM	54	0.6		
LY 400nM	19.6	1	0.000004	<0.001
SO 40nM	51.55	0.14	$0.00	<0.001
LY 400nM/SO 40nM	74.43333	1.2		
LY 800nM	43.6	1.1	0.000006	
SO 80nM	62.07667	1.78	0.000244	
LY 800nM/SO 80nM	86.3	0.8		
